# Supplementary material for: Scikick: A sidekick for workflow clarity and reproducibility during extensive data analysis
Source: PLoS One. 2023 Jul 27;18(7):e0289171. doi: 10.1371/journal.pone.0289171 (PMC10374128; doi:10.1371/journal.pone.0289171)
Supplement: S1 File — (ZIP) [file pone.0289171.s001.zip › scikick/README.html]

README


# PLOS ONE Supplementary Material

This supplementary code archive contains the source code for Scikick
v0.2.1. Also contained is the final product of the referenced scRNAseq
demonstration analysis which can be viewed by opening
`docs/scikick_documentation/single-cell_analysis/report/out_html/index.html`
in a web browser.

The latest code and documentation can be found at https://github.com/matthewcarlucci/scikick.

Scikick 0.2.1 README.md contents are shown below.

# Scikick - Notebook-Centric Analysis Workflows

## Overview

Collections of computational notebooks often lose coherence during
complex and branching investigations. Scikick is a command line utility
for managing ensembles of computational notebooks developed throughout a
project by providing simple commands for workflow configuration, report
generation, and state management.

---

## Getting Started

Read
the introduction for further details and installation
requirements.

See the tutorial
for a simple example usage of Scikick.

View
a data analysis report generated by Scikick.

Use the issue board
to provide feedback on Scikick (feature requests, bugs, and further
software-related comments).
