# Supplementary material for: Scikick: A sidekick for workflow clarity and reproducibility during extensive data analysis
Source: PLoS One. 2023 Jul 27;18(7):e0289171. doi: 10.1371/journal.pone.0289171 (PMC10374128; doi:10.1371/journal.pone.0289171)
Supplement: S1 File — (ZIP) [file pone.0289171.s001.zip › scikick/docs/scikick_documentation/index.html]

Scikick


# Scikick

A Companion for Notebook-Centric Scientific Practice

Start using Scikick 
  View a report generated by Scikick

Collections of computational notebooks often lose coherence during complex and branching investigations. Scikick is a command line utility for managing ensembles of computational notebooks. Scikick provides simple commands for setting the order of execution of scripts and notebooks to generate transparent analysis archives and manage the state of the project.
