# Supplementary material for: Scikick: A sidekick for workflow clarity and reproducibility during extensive data analysis
Source: PLoS One. 2023 Jul 27;18(7):e0289171. doi: 10.1371/journal.pone.0289171 (PMC10374128; doi:10.1371/journal.pone.0289171)
Supplement: S1 File — (ZIP) [file pone.0289171.s001.zip › scikick/docs/scikick_documentation/single-cell_analysis/report/out_html/index.html]

Project Overview


Single-cell Analysis

- Nestorowa
  - Import
  - Quality Control
  - Normalization
  - Further Exploration
- Grun
  - Import
  - Quality Control
  - Normalization
  - Further Exploration
- Paul
  - Import
  - Quality Control
  - Normalization
  - Further Exploration
- Merged
  - Merge
  - Combined Analysis

Code 

- Show All Code
- Hide All Code

# Project Overview

#### 17 February 2023

This project implements analyses from the OSCA
book on hematopoetic stem cell (HSC) single-cell RNA sequencing (scRNAseq) datasets. The main purpose of the project is to illustrate how Scikick projects are configured and typically used in a realistic setting. A walkthrough documents how Scikick was used throughout the project to execute and manage the state of the workflow.

# Credits to the Original OSCA Work

All pages in this project (except this one) were obtained directly from the source code of the OSCA book (https://github.com/Bioconductor/OrchestratingSingleCellAnalysis/, revision d56676f9d) released under a CC BY 4.0 license and created by: Robert Amezquita, Aaron Lun, Stephanie Hicks, and Raphael Gottardo.

Changes to the original source code are as follows:

- Notebooks were split across multiple notebooks and placed into directories to demonstrate the inter-notebook state management of Scikick.
- The first chunk of each original notebook was removed.
- Titles were added to each page.
- Headers were adjusted to the new document hierarchy.
- “Session Info” sections were removed from each page (replaced by Scikick outputs).
- The cache-based build system was replaced with standard read/write operations.
- Package loads were added to each page as needed.
- Referencing using functionality specific to the bookdown format was removed or replaced.

# Reading a Scikick Report

- Pages each correspond to a computational notebook.
- Notebooks were configured to execute in a specific order.
- The “Project Map” at the bottom of each page illustrates the order in which notebooks were executed.

## Project Map

The “Project Map” automatically generated by Scikick below (and at the bottom of all pages)
can aid in navigating and visualizing the project. Each node is a page that can be clicked to navigate to it. Pages can also be navigated with the standard bootstrap navigation at the top of each page.

---


Project History

**Project History**

**List of Contributors**

| Author | Number of Commits |
| --- | --- |
| Matthew Carlucci | 78 |
| Tadas Bareikis | 45 |
| tadasbar | 6 |

**Full List of Project Changes**

| Author | Message | Date | SHA |
| --- | --- | --- | --- |
| Matthew Carlucci | `Merge branch 'master' of github.com:matthewcarlucci/scikick` | 2023-02-17 21:37:09 GMT | a64b817b |
| Matthew Carlucci | `Update docs with real simulated changes instead of touch` | 2023-02-17 21:37:01 GMT | f59eb45d |
| Matthew Carlucci | `Version 0.2.1` | 2023-02-17 19:41:36 GMT | f88741e7 |
| Matthew Carlucci | `Always include a benchmark directory to satisfy snakemake wildcard rules` | 2023-02-17 19:28:08 GMT | 332a0bf8 |
| Matthew Carlucci | `Update touch tests which snakemake no longer responds to` | 2023-02-17 19:18:10 GMT | e8864634 |
| Matthew Carlucci | `Update tests to run with pytest and unittest to replace broken nose` | 2023-02-14 18:13:53 GMT | 84ff23b7 |
| Matthew Carlucci | `Bug fix for failing custom reportdir usage` | 2022-01-12 21:26:10 GMT | 5e23effe |
| Matthew Carlucci | `Add ignored header HTML content` | 2021-03-30 16:55:20 GMT | 72dc022e |
| Matthew Carlucci | `Version 0.2.0` | 2021-03-30 14:29:55 GMT | fbb1f63e |
| Matthew Carlucci | `Make error logging robust to missing log file` | 2021-03-29 20:38:54 GMT | ec432abb |
| Matthew Carlucci | `Add graphviz dependency for project maps` | 2021-03-29 20:38:02 GMT | ca7a8367 |
| Matthew Carlucci | `Update documentation with more details on robust archival of scRNAseq` | 2021-03-29 20:37:10 GMT | 46825577 |
| Matthew Carlucci | `Fix change to site generation to re-allow homepage-only sites` | 2021-03-22 22:28:17 GMT | d5fd890a |
| Matthew Carlucci | `Finish fixes to layout subdir usage` | 2021-03-19 15:32:43 GMT | 6b003b2f |
| Matthew Carlucci | `Use default retina settings for knitr to allow fig.cap usage, docs/figure update...` | 2021-03-18 19:00:55 GMT | 4b850a05 |
| Matthew Carlucci | `Middle of refactoring sk layout to fix bugs` | 2021-03-17 18:46:49 GMT | a30d711a |
| Matthew Carlucci | `Fix interactive plot handling` | 2021-03-16 21:28:56 GMT | c080c26b |
| Matthew Carlucci | `Fixes to project map subgraph assignments` | 2021-03-16 21:13:51 GMT | 24de163c |
| Matthew Carlucci | `New docs homepage and related README/setup changes` | 2021-03-16 21:11:14 GMT | ab51effe |
| Matthew Carlucci | `Addition of tutorial and documentation content with some minor code adjustments ...` | 2021-03-07 14:53:57 GMT | b30e1fb0 |
| Matthew Carlucci | `Remove or reformat references` | 2021-03-03 14:50:26 GMT | 1d751129 |
| Matthew Carlucci | `Add I/O commands to make project fully executable` | 2021-03-01 15:42:03 GMT | 98d53616 |
| Matthew Carlucci | `Split analysis into multiple notebooks (original notebooks are from revision d56...` | 2021-02-25 22:39:15 GMT | c9bbf31b |
| Matthew Carlucci | `Import select content from https://github.com/Bioconductor/OrchestratingSingleCe...` | 2021-02-25 20:33:33 GMT | d39f3b42 |
| Matthew Carlucci | `Add two tests for ipynb` | 2021-02-19 17:19:21 GMT | b8aa240b |
| Matthew Carlucci | `Add sk run --quiet and other minor fixes` | 2021-02-19 17:16:39 GMT | da422971 |
| Matthew Carlucci | `Refactor to have snakemake workflows in a more conventional format` | 2021-02-17 16:32:56 GMT | 27731059 |
| Matthew Carlucci | `Changes and fixes to messaging` | 2021-02-09 15:06:38 GMT | 28d8f8b1 |
| Matthew Carlucci | `Add an intermediate post markdown step to insert a project map to each page` | 2021-01-29 19:55:58 GMT | 7cabf903 |
| Matthew Carlucci | `Allow md files to depend on other steps` | 2021-01-26 16:46:44 GMT | 4688ed4f |
| Matthew Carlucci | `Fix issue determining site.yml files for an index only directory` | 2021-01-26 16:43:48 GMT | dcea9dfe |
| Matthew Carlucci | `Bug fixes in logging and sk mv` | 2021-01-12 21:42:10 GMT | 3a6cb532 |
| Matthew Carlucci | `Documentation and minor bug fixes for next release` | 2021-01-07 18:35:06 GMT | a37b1a1f |
| Matthew Carlucci | `Include system index file (homepage) in sk status` | 2021-01-06 16:33:01 GMT | 5203f613 |
| Matthew Carlucci | `Simplify code execution stack for R and Rmd by keeping log files and using snake...` | 2021-01-06 16:19:10 GMT | c186c213 |
| Matthew Carlucci | `Migrate layout setting code to own module` | 2021-01-04 18:19:26 GMT | ab7ccdfc |
| Matthew Carlucci | `Minor changes to init code` | 2021-01-04 18:15:07 GMT | 5dd90e7b |
| Matthew Carlucci | `Aggregate workflow properties and conveniece methods into ScikickConfig` | 2021-01-04 16:10:58 GMT | a4486415 |
| Matthew Carlucci | `Split status parsing by job type and directly use marker priroritization` | 2021-01-04 15:57:27 GMT | 44f635fe |
| Matthew Carlucci | `Minimized render site complexity and allow for custom outputs` | 2020-12-29 17:22:09 GMT | 56ead137 |
| Matthew Carlucci | `Further modularize Snakemake workflow and related scripts` | 2020-12-29 17:21:00 GMT | c86afb77 |
| Matthew Carlucci | `More modular and robust snakemake config setup` | 2020-12-17 22:21:57 GMT | 74824108 |
| Matthew Carlucci | `Minor modification to demo and template contents` | 2020-12-14 20:04:54 GMT | 0dbdc007 |
| Matthew Carlucci | `Misc other related to recent changes` | 2020-12-11 14:56:31 GMT | 0c9599bc |
| Matthew Carlucci | `sk status: fix test and rewrite snake status` | 2020-12-10 22:14:07 GMT | a9cafb15 |
| Matthew Carlucci | `Modify added page content, more robustness to commit special characters, include...` | 2020-12-10 22:03:19 GMT | 7dad100d |
| Matthew Carlucci | `Rewrite sk config` | 2020-12-10 21:57:01 GMT | 540036e0 |
| Matthew Carlucci | `Internal: rely on skconfig more during sk run and sk config` | 2020-12-10 21:49:19 GMT | 9e15b60a |
| Matthew Carlucci | `Misc refactor and commenting` | 2020-12-07 20:30:20 GMT | 397c95a6 |
| Matthew Carlucci | `Remove body width CSS` | 2020-12-03 16:34:22 GMT | 07d736ec |
| Matthew Carlucci | `Allow spaces in project path` | 2020-12-01 22:31:56 GMT | 32b1ef1e |
| Matthew Carlucci | `README update with demo gif` | 2020-12-01 14:33:14 GMT | 8665b5a5 |
| Matthew Carlucci | `New sk init template setting analysis as empty dict` | 2020-11-27 17:03:45 GMT | 3cfbe5c6 |
| Matthew Carlucci | `Update sk del with new -d usage for issue #2 (multiple -d)` | 2020-11-27 17:02:12 GMT | a98f5411 |
| Matthew Carlucci | `Updating parsers to use script instead of rmd` | 2020-11-27 17:00:47 GMT | 9232007c |
| Matthew Carlucci | `Modify demo for new sk add usage and add a demo test` | 2020-11-27 16:55:17 GMT | c3422bd7 |
| Matthew Carlucci | `Modifications to sk add to address part of issue #2 (multiple -d)` | 2020-11-27 16:53:41 GMT | 93007d06 |
| Matthew Carlucci | `Add argument need pages to yaml in() for steps requiring non-empty analysis` | 2020-11-27 16:47:25 GMT | 156b9179 |
| Matthew Carlucci | `Dump snakemake logs after unexpected errors` | 2020-11-23 19:45:47 GMT | 2a863837 |
| Matthew Carlucci | `Code cleanup and commenting` | 2020-11-23 16:32:01 GMT | e01f0977 |
| Matthew Carlucci | `Allow sk run <doc> usage with empty yaml` | 2020-10-01 16:03:34 GMT | 5ec45662 |
| Matthew Carlucci | `Change analysis metadata from button to details tag` | 2020-10-01 16:02:22 GMT | d898a354 |
| Matthew Carlucci | `Code cleanup (few functional differences)` | 2020-10-01 15:49:36 GMT | 026e863f |
| Matthew Carlucci | `Internal function name changes and sk init description update` | 2020-09-29 20:59:42 GMT | 52fbc636 |
| Matthew Carlucci | `Modify defaults allow execution of run snakemake without arguments` | 2020-09-28 19:59:20 GMT | 0c950981 |
| Matthew Carlucci | `Simplify software check outputs` | 2020-09-28 19:57:43 GMT | 63ac1ef5 |
| Matthew Carlucci | `Clean up sk init and demo outputs` | 2020-09-28 19:04:56 GMT | 5aa4446a |
| Matthew Carlucci | `Include a snakemake badge` | 2020-09-28 19:03:59 GMT | 81716a03 |
| Matthew Carlucci | `Add pop snakefile for debugging scikick Snakefile issues` | 2020-09-25 16:16:37 GMT | 42bf8e6a |
| Matthew Carlucci | `Rename rules and reorganize scripts` | 2020-09-25 16:14:31 GMT | c6a65140 |
| tadasbar | `Merge branch 'dev' of https://github.com/matthewcarlucci/scikick into dev` | 2020-09-10 13:53:51 GMT | 0dedc510 |
| tadasbar | `Updated the comments in scikick/status.py` | 2020-09-10 08:04:59 GMT | 729e5106 |
| Matthew Carlucci | `Increment to 0.1.2 for new release` | 2020-09-09 16:28:20 GMT | 1c62fbb7 |
| tadasbar | `Limit the "Unrecognized scikick.yml field" message to sk (run, config, status)` | 2020-09-09 12:18:32 GMT | 20b23090 |
| Tadas Bareikis | `Change help string for "sk status rmd"` | 2020-09-04 02:12:01 GMT | 5bf396f5 |
| Matthew Carlucci | `Update changelog for nearing 0.1.2 release` | 2020-09-03 21:34:47 GMT | 4402fe56 |
| Matthew Carlucci | `Include more installation details with a conda install method` | 2020-09-03 21:33:12 GMT | f581b8aa |
| Tadas Bareikis | `Update "sk status --help"; Change "-e-", "-u-" definitions and output in "sk sta...` | 2020-09-03 10:50:39 GMT | 71468f38 |
| Tadas Bareikis | `Print warnings/errors only in case of error is non-verbose output of "sk run"` | 2020-09-03 09:00:26 GMT | 31684b39 |
| Tadas Bareikis | `Add "-u-" marker for "sk status"` | 2020-09-02 04:50:54 GMT | 73d023fb |
| Tadas Bareikis | `Print snakemake logfile name even in case of error; fix scikick.yml field check ...` | 2020-09-02 03:04:00 GMT | d3064381 |
| Tadas Bareikis | `Check for unsupported scikick.yml fields on every scikick execution` | 2020-09-01 08:11:23 GMT | 8c358568 |
| Tadas Bareikis | `Change "sk status" output by adding additional info to "m--" marker` | 2020-09-01 06:02:41 GMT | 9e006504 |
| Tadas Bareikis | `Add knitmeta test for singularity run` | 2020-08-28 03:43:26 GMT | 12978314 |
| Tadas Bareikis | `Add singularity (and conda) support for pageRender rule` | 2020-08-28 03:27:23 GMT | a4ad452e |
| Tadas Bareikis | `Modify messages for internal/external dependencies` | 2020-08-27 07:36:47 GMT | f8dafa31 |
| Tadas Bareikis | `Make "sk config" print all variables when no options are provided` | 2020-08-27 06:34:40 GMT | a04b4d3f |
| Tadas Bareikis | `Add messages for internal/external dependencies` | 2020-08-27 04:36:51 GMT | e297a12d |
| Tadas Bareikis | `Add warnings for unsupported formats in scikick.yml` | 2020-08-27 02:49:32 GMT | e6a79c25 |
| Tadas Bareikis | `Merge branch 'dev' of https://github.com/matthewcarlucci/scikick into dev` | 2020-08-26 11:03:14 GMT | 201f3b29 |
| Tadas Bareikis | `Fix "sk status" output for modified md files` | 2020-08-26 11:02:38 GMT | e71ed31d |
| Tadas Bareikis | `Add commit sha column to the "List of Changes" in the footer` | 2020-08-26 05:51:47 GMT | 6b015c03 |
| Tadas Bareikis | `Minor fix in "sk status" output for internal dependencies` | 2020-08-26 04:36:54 GMT | 56467e9d |
| Matthew Carlucci | `Merge branch 'dev' of github.com:matthewcarlucci/scikick into dev` | 2020-08-25 14:13:28 GMT | 63e2729c |
| Matthew Carlucci | `Add CHANGELOG.md` | 2020-08-25 14:13:18 GMT | 1061588d |
| Matthew Carlucci | `Update sk init --readme output` | 2020-08-25 14:10:26 GMT | 06012c0f |
| Tadas Bareikis | `Fix "sk status" output for missing htmls` | 2020-08-22 12:46:47 GMT | ed2b21fb |
| Tadas Bareikis | `Change the version from "0.1.1" to "0.1.1.dev0"` | 2020-08-21 14:40:58 GMT | 9982e6e4 |
| Tadas Bareikis | `Retain timestamp of md when correcting for moved figuredir` | 2020-08-20 18:24:25 GMT | 4d426f19 |
| Tadas Bareikis | `Add snakemake's "target" argument to "sk run" as "rmds"` | 2020-08-20 12:24:11 GMT | 8a5dd12b |
| Tadas Bareikis | `Add the option to specify an rmd for "sk status"` | 2020-08-18 14:44:10 GMT | 632985cb |
| Tadas Bareikis | `Ensure that mandatory fields (analysis and reportdir) are present in the yaml` | 2020-08-18 11:50:48 GMT | 1497f5ac |
| Tadas Bareikis | `Warn and move on after git failures in 'sk mv -g'` | 2020-08-18 11:02:23 GMT | 0df47142 |
| Tadas Bareikis | `Add error messages on snakemake failures in 'sk run'` | 2020-08-17 17:00:03 GMT | 6d9e3cd0 |
| Tadas Bareikis | `Add "--copy-deps" argument to "sk add"` | 2020-08-17 14:07:25 GMT | e6976008 |
| Tadas Bareikis | `Fix "Generating * html" output` | 2020-08-17 11:17:34 GMT | 430584ed |
| Tadas Bareikis | `Fix 'sk mv' for non-Rmd dependencies` | 2020-08-16 22:02:55 GMT | 7db5e63f |
| Tadas Bareikis | `Fix R package renv usage with sk run` | 2020-08-14 13:46:50 GMT | a07b920b |
| Tadas Bareikis | `--singularity-args can be used with the -s argument` | 2020-08-13 18:11:35 GMT | d71bd937 |
| tadasbar | `Remove gitpython dependency, use R's git2r instead` | 2020-08-13 14:32:02 GMT | 7b910597 |
| tadasbar | `Fix Git Log in footer for non-Rmd files` | 2020-08-07 09:16:07 GMT | 17475d5e |
| tadasbar | `Add checks for git2r version` | 2020-08-06 12:57:44 GMT | df743c18 |
| Tadas Bareikis | `Rewrite the tests` | 2020-08-04 07:50:46 GMT | af60b461 |
| Tadas Bareikis | `Merge branch 'master' into dev` | 2020-08-04 02:01:58 GMT | a3b63679 |
| Tadas Bareikis | `Update the setup.py file and push to pip v0.1.1` | 2020-07-28 08:56:53 GMT | d405da9e |
| Tadas Bareikis | `Merge branch 'dev' of https://github.com/matthewcarlucci/scikick into dev` | 2020-07-28 05:02:44 GMT | 07dfbe15 |
| Tadas Bareikis | `Added sk init --demo and fixed index page usage` | 2020-07-28 03:50:56 GMT | 5e6180b3 |
| Tadas Bareikis | `Appending the system index.Rmd to index pages` | 2020-07-27 10:59:52 GMT | b0669d7f |
| Tadas Bareikis | `Index page fixes: index page at the root; no error when adding a 3rd... index pa...` | 2020-07-27 08:52:53 GMT | 9e704ff5 |
| Tadas Bareikis | `Removed 'plot.Rmd' from the demo` | 2020-07-27 08:19:19 GMT | 225db271 |
| Tadas Bareikis | `Updated the demo` | 2020-07-27 02:50:41 GMT | 725db621 |
| Tadas Bareikis | `Changed some wording in the demo` | 2020-07-26 12:09:23 GMT | d8e6519e |
| Tadas Bareikis | `Added 'sk init --demo'` | 2020-07-26 11:59:30 GMT | 409d5b77 |
| Tadas Bareikis | `Removed unnecessary outputs` | 2020-07-26 06:01:27 GMT | 19a2cd2a |
| Tadas Bareikis | `Fixes in 'sk status' for the index page` | 2020-07-26 05:47:06 GMT | 77658c59 |
| Matthew Carlucci | `Update README.md introduction` | 2020-07-24 19:15:32 GMT | 8dc6c7b0 |
| Tadas Bareikis | `Silenced git warnings, using git2r in template index.Rmd` | 2020-07-24 13:30:41 GMT | 450685c9 |
| Tadas Bareikis | `New user-defined index page usage` | 2020-07-24 12:25:00 GMT | 0725b105 |
| Matthew Carlucci | `Initial release` | 2020-07-23 22:07:27 GMT | 968ff086 |

  
  


Next (Project Map)


skmap


cluster\_/

/


cluster\_notebooks/nestorowa/

notebooks/nestorowa/


cluster\_notebooks/grun/

notebooks/grun/


cluster\_notebooks/paul/

notebooks/paul/


cluster\_notebooks/merged/

notebooks/merged/


notebooks/grun/quality\_control.Rmd


Quality Control


notebooks/merged/merge.Rmd


Merge


notebooks/grun/quality\_control.Rmd->notebooks/merged/merge.Rmd


notebooks/grun/normalization.Rmd


Normalization


notebooks/grun/quality\_control.Rmd->notebooks/grun/normalization.Rmd


notebooks/merged/combined\_analysis.Rmd


Combined Analysis


notebooks/merged/merge.Rmd->notebooks/merged/combined\_analysis.Rmd


notebooks/paul/quality\_control.Rmd


Quality Control


notebooks/paul/quality\_control.Rmd->notebooks/merged/merge.Rmd


notebooks/paul/normalization.Rmd


Normalization


notebooks/paul/quality\_control.Rmd->notebooks/paul/normalization.Rmd


notebooks/nestorowa/normalization.Rmd


Normalization


notebooks/nestorowa/normalization.Rmd->notebooks/merged/merge.Rmd


notebooks/nestorowa/further\_exploration.Rmd


Further Exploration


notebooks/nestorowa/normalization.Rmd->notebooks/nestorowa/further\_exploration.Rmd


index.Rmd


Index


notebooks/nestorowa/import.Rmd


Import


notebooks/nestorowa/quality\_control.Rmd


Quality Control


notebooks/nestorowa/import.Rmd->notebooks/nestorowa/quality\_control.Rmd


notebooks/nestorowa/quality\_control.Rmd->notebooks/nestorowa/normalization.Rmd


notebooks/grun/import.Rmd


Import


notebooks/grun/import.Rmd->notebooks/grun/quality\_control.Rmd


notebooks/grun/further\_exploration.Rmd


Further Exploration


notebooks/grun/normalization.Rmd->notebooks/grun/further\_exploration.Rmd


notebooks/paul/import.Rmd


Import


notebooks/paul/import.Rmd->notebooks/paul/quality\_control.Rmd


notebooks/paul/further\_exploration.Rmd


Further Exploration


notebooks/paul/normalization.Rmd->notebooks/paul/further\_exploration.Rmd


---
