# Supplementary material for: Scikick: A sidekick for workflow clarity and reproducibility during extensive data analysis
Source: PLoS One. 2023 Jul 27;18(7):e0289171. doi: 10.1371/journal.pone.0289171 (PMC10374128; doi:10.1371/journal.pone.0289171)
Supplement: S1 File — (ZIP) [file pone.0289171.s001.zip › scikick/docs/scikick_documentation/single-cell_analysis/report/out_html/notebooks/further_exploration.html]

Further Exploration


Single-cell Analysis

- Import
- Quality Control
- Normalization
- Further Exploration

Code 

- Show All Code
- Hide All Code

# Further Exploration

#### 17 February 2023

```
library(scater)
library(scran)
library(BiocStyle)
library(pheatmap)
sce.nest <- readRDS("output/nestorowa_normalization_sce.RDS")
top.nest <- readRDS("output/nestorowa_normalization_top.RDS")
dec.nest <- readRDS("output/nestorowa_normalization_dec.RDS")
```

# Dimensionality reduction

```
set.seed(101010011)
sce.nest <- denoisePCA(sce.nest, technical=dec.nest, subset.row=top.nest)
sce.nest <- runTSNE(sce.nest, dimred="PCA")
```

We check that the number of retained PCs is sensible.

```
ncol(reducedDim(sce.nest, "PCA"))
```

```
[1] 9
```

# Clustering

```
snn.gr <- buildSNNGraph(sce.nest, use.dimred="PCA")
colLabels(sce.nest) <- factor(igraph::cluster_walktrap(snn.gr)$membership)
```

```
table(colLabels(sce.nest))
```

```
  1   2   3   4   5   6   7   8   9 
203 472 258 175 142 229  20  83  74
```

```
plotTSNE(sce.nest, colour_by="label")
```

Obligatory \(t\)-SNE plot of the Nestorowa HSC dataset, where each point represents a cell and is colored according to the assigned cluster.

# Marker gene detection

```
markers <- findMarkers(sce.nest, colLabels(sce.nest), 
    test.type="wilcox", direction="up", lfc=0.5,
    row.data=rowData(sce.nest)[,"SYMBOL",drop=FALSE])
```

To illustrate the manual annotation process, we examine the marker genes for one of the clusters.
Upregulation of *Car2*, *Hebp1* amd hemoglobins indicates that cluster 8 contains erythroid precursors.

```
chosen <- markers[['8']]
best <- chosen[chosen$Top <= 10,]
aucs <- getMarkerEffects(best, prefix="AUC")
rownames(aucs) <- best$SYMBOL

library(pheatmap)
pheatmap(aucs, color=viridis::plasma(100))
```

Heatmap of the AUCs for the top marker genes in cluster 8 compared to all other clusters.

# Cell type annotation

```
library(SingleR)
mm.ref <- MouseRNAseqData()

# Renaming to symbols to match with reference row names.
renamed <- sce.nest
rownames(renamed) <- uniquifyFeatureNames(rownames(renamed),
    rowData(sce.nest)$SYMBOL)
labels <- SingleR(renamed, mm.ref, labels=mm.ref$label.fine)
```

Most clusters are not assigned to any single lineage, which is perhaps unsurprising given that HSCs are quite different from their terminal fates.
Cluster 8 is considered to contain erythrocytes, which is roughly consistent with our conclusions from the marker gene analysis above.

```
tab <- table(labels$labels, colLabels(sce.nest))
pheatmap(log10(tab+10), color=viridis::viridis(100))
```

Heatmap of the distribution of cells for each cluster in the Nestorowa HSC dataset, based on their assignment to each label in the mouse RNA-seq references from the *SingleR* package.

# Miscellaneous analyses

This dataset also contains information about the protein abundances in each cell from FACS.
There is barely any heterogeneity in the chosen markers across the clusters;
this is perhaps unsurprising given that all cells should be HSCs of some sort.

```
Y <- colData(sce.nest)$FACS
keep <- rowSums(is.na(Y))==0 # Removing NA intensities.

se.averaged <- sumCountsAcrossCells(t(Y[keep,]), 
    colLabels(sce.nest)[keep], average=TRUE)
averaged <- assay(se.averaged)

log.intensities <- log2(averaged+1)
centered <- log.intensities - rowMeans(log.intensities)
pheatmap(centered, breaks=seq(-1, 1, length.out=101))
```

Heatmap of the centered log-average intensity for each target protein quantified by FACS in the Nestorowa HSC dataset.


---


Click to see page metadata

Computation Started: `2023-02-17 16:44:17`

Finished in `44.12 secs`

---

**Git Log**

No git history available for this page

---

**Packages**

| package | version | date |
| --- | --- | --- |
| Rtsne | 0.15 | 2020-07-15 |
| ggbeeswarm | 0.6.0 | 2020-07-16 |
| colorspace | 2.0-0 | 2020-11-12 |
| ellipsis | 0.3.1 | 2020-07-15 |
| scuttle | 1.0.4 | 2020-12-18 |
| bluster | 1.0.0 | 2020-10-28 |
| XVector | 0.30.0 | 2020-10-29 |
| GenomicRanges | 1.42.0 | 2020-10-28 |
| BiocNeighbors | 1.8.2 | 2020-12-08 |
| farver | 2.0.3 | 2020-07-15 |
| stats | 4.0.1 | 2020-06-07 |
| bit64 | 4.0.5 | 2020-08-31 |
| interactiveDisplayBase | 1.28.0 | 2020-10-28 |
| AnnotationDbi | 1.52.0 | 2020-10-28 |
| fansi | 0.4.2 | 2021-01-16 |
| sparseMatrixStats | 1.2.0 | 2020-10-28 |
| cachem | 1.0.3 | 2021-02-05 |
| knitr | 1.30 | 2020-09-23 |
| scater | 1.18.3 | 2020-11-09 |
| base | 4.0.1 | 2020-06-07 |
| dbplyr | 2.1.1 | 2021-04-07 |
| pheatmap | 1.0.12 | 2020-07-16 |
| shiny | 1.5.0 | 2020-07-16 |
| BiocManager | 1.30.10 | 2020-07-15 |
| compiler | 4.0.1 | 2020-06-07 |
| httr | 1.4.2 | 2020-07-23 |
| dqrng | 0.2.1 | 2020-07-15 |
| assertthat | 0.2.1 | 2020-07-15 |
| Matrix | 1.2-18 | 2020-06-07 |
| fastmap | 1.0.1 | 2020-07-15 |
| limma | 3.46.0 | 2020-10-28 |
| later | 1.1.0.1 | 2020-07-15 |
| BiocSingular | 1.6.0 | 2020-10-28 |
| htmltools | 0.5.1 | 2021-01-13 |
| tools | 4.0.1 | 2020-06-07 |
| rsvd | 1.0.3 | 2020-07-15 |
| igraph | 1.2.6 | 2020-10-07 |
| gtable | 0.3.0 | 2020-07-15 |
| glue | 1.4.2 | 2020-08-28 |
| GenomeInfoDbData | 1.2.4 | 2020-11-03 |
| dplyr | 1.0.5 | 2021-03-06 |
| grDevices | 4.0.1 | 2020-06-07 |
| rappdirs | 0.3.1 | 2020-07-15 |
| Rcpp | 1.0.6 | 2021-01-16 |
| Biobase | 2.50.0 | 2020-10-28 |
| vctrs | 0.3.6 | 2020-12-18 |
| ExperimentHub | 1.16.0 | 2020-10-28 |
| DelayedMatrixStats | 1.12.2 | 2021-01-13 |
| xfun | 0.23 | 2021-05-16 |
| SingleR | 1.4.0 | 2020-10-28 |
| stringr | 1.4.0 | 2020-07-15 |
| beachmat | 2.6.4 | 2020-12-21 |
| mime | 0.9 | 2020-07-15 |
| lifecycle | 1.0.0 | 2021-02-16 |
| irlba | 2.3.3 | 2020-07-15 |
| statmod | 1.4.35 | 2020-10-20 |
| AnnotationHub | 2.22.0 | 2020-10-28 |
| edgeR | 3.32.1 | 2021-01-15 |
| zlibbioc | 1.36.0 | 2020-10-29 |
| scales | 1.1.1 | 2020-07-16 |
| BiocStyle | 2.18.1 | 2020-11-25 |
| graphics | 4.0.1 | 2020-06-07 |
| promises | 1.1.1 | 2020-07-16 |
| MatrixGenerics | 1.2.0 | 2020-10-28 |
| parallel | 4.0.1 | 2020-06-07 |
| SummarizedExperiment | 1.20.0 | 2020-10-28 |
| RColorBrewer | 1.1-2 | 2020-07-15 |
| utils | 4.0.1 | 2020-06-07 |
| SingleCellExperiment | 1.12.0 | 2020-10-28 |
| yaml | 2.2.1 | 2020-07-15 |
| curl | 4.3 | 2020-06-15 |
| memoise | 2.0.0 | 2021-01-27 |
| gridExtra | 2.3 | 2020-07-15 |
| ggplot2 | 3.3.3 | 2020-12-31 |
| datasets | 4.0.1 | 2020-06-07 |
| stringi | 1.5.3 | 2020-09-10 |
| RSQLite | 2.2.2 | 2021-01-09 |
| BiocVersion | 3.12.0 | 2020-05-15 |
| highr | 0.8 | 2020-07-15 |
| S4Vectors | 0.28.1 | 2020-12-10 |
| scran | 1.18.3 | 2020-12-22 |
| BiocGenerics | 0.36.0 | 2020-10-28 |
| BiocParallel | 1.24.1 | 2020-11-07 |
| GenomeInfoDb | 1.26.2 | 2020-12-09 |
| rlang | 0.4.10 | 2020-12-31 |
| pkgconfig | 2.0.3 | 2020-07-15 |
| matrixStats | 0.57.0 | 2020-09-26 |
| bitops | 1.0-6 | 2020-07-15 |
| evaluate | 0.14 | 2020-06-15 |
| lattice | 0.20-41 | 2020-06-07 |
| purrr | 0.3.4 | 2020-07-15 |
| labeling | 0.4.2 | 2020-10-21 |
| cowplot | 1.1.1 | 2020-12-31 |
| bit | 4.0.4 | 2020-08-04 |
| tidyselect | 1.1.0 | 2020-07-15 |
| magrittr | 2.0.1 | 2020-11-18 |
| R6 | 2.5.0 | 2020-10-29 |
| IRanges | 2.24.1 | 2020-12-13 |
| generics | 0.1.0 | 2020-11-01 |
| DelayedArray | 0.16.0 | 2020-10-28 |
| DBI | 1.1.1 | 2021-01-16 |
| pillar | 1.6.0 | 2021-04-14 |
| withr | 2.4.2 | 2021-04-19 |
| RCurl | 1.98-1.2 | 2020-07-15 |
| tibble | 3.1.1 | 2021-04-19 |
| crayon | 1.4.1 | 2021-02-09 |
| utf8 | 1.1.4 | 2020-07-15 |
| BiocFileCache | 1.14.0 | 2020-10-28 |
| rmarkdown | 2.8 | 2021-05-08 |
| viridis | 0.5.1 | 2020-07-17 |
| locfit | 1.5-9.4 | 2020-07-15 |
| grid | 4.0.1 | 2020-06-07 |
| git2r | 0.28.0 | 2021-01-11 |
| blob | 1.2.1 | 2020-07-15 |
| methods | 4.0.1 | 2020-06-07 |
| digest | 0.6.27 | 2020-10-25 |
| xtable | 1.8-4 | 2020-07-15 |
| httpuv | 1.5.5 | 2021-01-13 |
| stats4 | 4.0.1 | 2020-06-07 |
| munsell | 0.5.0 | 2020-07-15 |
| celldex | 1.0.0 | 2020-11-10 |
| beeswarm | 0.2.3 | 2020-07-15 |
| viridisLite | 0.3.0 | 2020-06-15 |
| vipor | 0.4.5 | 2020-07-15 |

---

**System Information**

|  | systemInfo |
| --- | --- |
| version | R version 4.0.1 (2020-06-06) |
| platform | x86\_64-apple-darwin17.0 (64-bit) |
| locale | en\_CA.UTF-8 |
| OS | macOS 10.16 |
| UI | X11 |

**Scikick Configuration**

```
cat scikick.yml
```

```
### Scikick Project Workflow Configuration File

# Directory where Scikick will store all standard notebook outputs
reportdir: report

# --- Content below here is best modified by using the Scikick CLI ---

# Notebook Execution Configuration (format summarized below)
# analysis:
#  first_notebook.Rmd:
#  second_notebook.Rmd: 
#  - first_notebook.Rmd     # must execute before second_notebook.Rmd
#  - functions.R            # file is used by second_notebook.Rmd
#
# Each analysis item is executed to generate md and html files, E.g.:
# 1. <reportdir>/out_md/first_notebook.md
# 2. <reportdir>/out_html/first_notebook.html
analysis: !!omap
- index.Rmd:
- notebooks/import.Rmd:
- notebooks/quality_control.Rmd:
  - notebooks/import.Rmd
- notebooks/normalization.Rmd:
  - notebooks/quality_control.Rmd
- notebooks/further_exploration.Rmd:
  - notebooks/normalization.Rmd
version_info:
  snakemake: 6.0.2
  ruamel.yaml: 0.16.12
  scikick: 0.2.1
# Optional site theme customization
output:
  BiocStyle::html_document:
    code_folding: hide
    theme: readable
    toc_float: true
    toc: true
    number_sections: false
    toc_depth: 5
    self_contained: true
```

---

**Functions**


  
  


Next (Project Map)


skmap


cluster\_/

/


cluster\_notebooks/

notebooks/


index.Rmd


Index


notebooks/import.Rmd


Import


notebooks/quality\_control.Rmd


Quality Control


notebooks/import.Rmd->notebooks/quality\_control.Rmd


notebooks/normalization.Rmd


Normalization


notebooks/quality\_control.Rmd->notebooks/normalization.Rmd


notebooks/further\_exploration.Rmd


Further Exploration


notebooks/normalization.Rmd->notebooks/further\_exploration.Rmd


---
