# Supplementary material for: Scikick: A sidekick for workflow clarity and reproducibility during extensive data analysis
Source: PLoS One. 2023 Jul 27;18(7):e0289171. doi: 10.1371/journal.pone.0289171 (PMC10374128; doi:10.1371/journal.pone.0289171)
Supplement: S1 File — (ZIP) [file pone.0289171.s001.zip › scikick/docs/scikick_documentation/single-cell_analysis/report/out_html/notebooks/paul/quality_control.html]

Quality Control


Single-cell Analysis

- Nestorowa
  - Import
  - Quality Control
  - Normalization
  - Further Exploration
- Grun
  - Import
  - Quality Control
  - Normalization
  - Further Exploration
- Paul
  - Import
  - Quality Control
  - Normalization
  - Further Exploration
- Merged
  - Merge
  - Combined Analysis

Code 

- Show All Code
- Hide All Code

# Quality Control

#### 17 February 2023

```
sce.paul <- readRDS("output/paul_import_sce.RDS")
```

```
unfiltered <- sce.paul
```

For some reason, only one mitochondrial transcripts are available, so we will perform quality control using only the library size and number of detected features.
Ideally, we would simply block on the plate of origin to account for differences in processing, but unfortunately, it seems that many plates have a large proportion (if not outright majority) of cells with poor values for both metrics.
We identify such plates based on the presence of very low outlier thresholds, for some arbitrary definition of “low”; we then redefine thresholds using information from the other (presumably high-quality) plates.

```
library(scater)
stats <- perCellQCMetrics(sce.paul) 
qc <- quickPerCellQC(stats, batch=sce.paul$Plate_ID)

# Detecting batches with unusually low threshold values.
lib.thresholds <- attr(qc$low_lib_size, "thresholds")["lower",]
nfeat.thresholds <- attr(qc$low_n_features, "thresholds")["lower",]
ignore <- union(names(lib.thresholds)[lib.thresholds < 100],
    names(nfeat.thresholds)[nfeat.thresholds < 100])

# Repeating the QC using only the "high-quality" batches.
qc2 <- quickPerCellQC(stats, batch=sce.paul$Plate_ID,
    subset=!sce.paul$Plate_ID %in% ignore)
sce.paul <- sce.paul[,!qc2$discard]
```

We examine the number of cells discarded for each reason.

```
colSums(as.matrix(qc2))
```

```
  low_lib_size low_n_features        discard 
          1695           1781           1783
```

We create some diagnostic plots for each metric.

```
colData(unfiltered) <- cbind(colData(unfiltered), stats)
unfiltered$discard <- qc2$discard
unfiltered$Plate_ID <- factor(unfiltered$Plate_ID)

gridExtra::grid.arrange(
    plotColData(unfiltered, y="sum", x="Plate_ID", colour_by="discard") +
        scale_y_log10() + ggtitle("Total count"),
    plotColData(unfiltered, y="detected", x="Plate_ID", colour_by="discard") +
        scale_y_log10() + ggtitle("Detected features"),
    ncol=1
)
```

Distribution of each QC metric across cells in the Paul HSC dataset. Each point represents a cell and is colored according to whether that cell was discarded.

```
saveRDS(sce.paul,"output/paul_quality_control_sce.RDS")
```


---


Click to see page metadata

Computation Started: `2023-02-17 16:45:48`

Finished in `15.077 secs`

---

**Git Log**

No git history available for this page

---

**Packages**

| package | version | date |
| --- | --- | --- |
| Rcpp | 1.0.6 | 2021-01-16 |
| rsvd | 1.0.3 | 2020-07-15 |
| lattice | 0.20-41 | 2020-06-07 |
| digest | 0.6.27 | 2020-10-25 |
| assertthat | 0.2.1 | 2020-07-15 |
| SingleCellExperiment | 1.12.0 | 2020-10-28 |
| utf8 | 1.1.4 | 2020-07-15 |
| R6 | 2.5.0 | 2020-10-29 |
| GenomeInfoDb | 1.26.2 | 2020-12-09 |
| stats4 | 4.0.1 | 2020-06-07 |
| evaluate | 0.14 | 2020-06-15 |
| highr | 0.8 | 2020-07-15 |
| ggplot2 | 3.3.3 | 2020-12-31 |
| pillar | 1.6.0 | 2021-04-14 |
| sparseMatrixStats | 1.2.0 | 2020-10-28 |
| utils | 4.0.1 | 2020-06-07 |
| zlibbioc | 1.36.0 | 2020-10-29 |
| rlang | 0.4.10 | 2020-12-31 |
| irlba | 2.3.3 | 2020-07-15 |
| S4Vectors | 0.28.1 | 2020-12-10 |
| Matrix | 1.2-18 | 2020-06-07 |
| BiocNeighbors | 1.8.2 | 2020-12-08 |
| BiocParallel | 1.24.1 | 2020-11-07 |
| stringr | 1.4.0 | 2020-07-15 |
| RCurl | 1.98-1.2 | 2020-07-15 |
| munsell | 0.5.0 | 2020-07-15 |
| beachmat | 2.6.4 | 2020-12-21 |
| DelayedArray | 0.16.0 | 2020-10-28 |
| compiler | 4.0.1 | 2020-06-07 |
| vipor | 0.4.5 | 2020-07-15 |
| BiocSingular | 1.6.0 | 2020-10-28 |
| xfun | 0.23 | 2021-05-16 |
| pkgconfig | 2.0.3 | 2020-07-15 |
| stats | 4.0.1 | 2020-06-07 |
| BiocGenerics | 0.36.0 | 2020-10-28 |
| ggbeeswarm | 0.6.0 | 2020-07-16 |
| tidyselect | 1.1.0 | 2020-07-15 |
| SummarizedExperiment | 1.20.0 | 2020-10-28 |
| tibble | 3.1.1 | 2021-04-19 |
| gridExtra | 2.3 | 2020-07-15 |
| GenomeInfoDbData | 1.2.4 | 2020-11-03 |
| IRanges | 2.24.1 | 2020-12-13 |
| matrixStats | 0.57.0 | 2020-09-26 |
| grDevices | 4.0.1 | 2020-06-07 |
| viridisLite | 0.3.0 | 2020-06-15 |
| fansi | 0.4.2 | 2021-01-16 |
| crayon | 1.4.1 | 2021-02-09 |
| dplyr | 1.0.5 | 2021-03-06 |
| withr | 2.4.2 | 2021-04-19 |
| bitops | 1.0-6 | 2020-07-15 |
| grid | 4.0.1 | 2020-06-07 |
| gtable | 0.3.0 | 2020-07-15 |
| lifecycle | 1.0.0 | 2021-02-16 |
| DBI | 1.1.1 | 2021-01-16 |
| git2r | 0.28.0 | 2021-01-11 |
| magrittr | 2.0.1 | 2020-11-18 |
| datasets | 4.0.1 | 2020-06-07 |
| scales | 1.1.1 | 2020-07-16 |
| stringi | 1.5.3 | 2020-09-10 |
| scuttle | 1.0.4 | 2020-12-18 |
| farver | 2.0.3 | 2020-07-15 |
| XVector | 0.30.0 | 2020-10-29 |
| viridis | 0.5.1 | 2020-07-17 |
| scater | 1.18.3 | 2020-11-09 |
| DelayedMatrixStats | 1.12.2 | 2021-01-13 |
| ellipsis | 0.3.1 | 2020-07-15 |
| graphics | 4.0.1 | 2020-06-07 |
| generics | 0.1.0 | 2020-11-01 |
| vctrs | 0.3.6 | 2020-12-18 |
| cowplot | 1.1.1 | 2020-12-31 |
| base | 4.0.1 | 2020-06-07 |
| tools | 4.0.1 | 2020-06-07 |
| Biobase | 2.50.0 | 2020-10-28 |
| glue | 1.4.2 | 2020-08-28 |
| beeswarm | 0.2.3 | 2020-07-15 |
| purrr | 0.3.4 | 2020-07-15 |
| MatrixGenerics | 1.2.0 | 2020-10-28 |
| parallel | 4.0.1 | 2020-06-07 |
| colorspace | 2.0-0 | 2020-11-12 |
| GenomicRanges | 1.42.0 | 2020-10-28 |
| knitr | 1.30 | 2020-09-23 |
| methods | 4.0.1 | 2020-06-07 |

---

**System Information**

|  | systemInfo |
| --- | --- |
| version | R version 4.0.1 (2020-06-06) |
| platform | x86\_64-apple-darwin17.0 (64-bit) |
| locale | en\_CA.UTF-8 |
| OS | macOS 10.16 |
| UI | X11 |

**Scikick Configuration**

```
cat scikick.yml
```

```
### Scikick Project Workflow Configuration File

# Directory where Scikick will store all standard notebook outputs
reportdir: report

# --- Content below here is best modified by using the Scikick CLI ---

# Notebook Execution Configuration (format summarized below)
# analysis:
#  first_notebook.Rmd:
#  second_notebook.Rmd: 
#  - first_notebook.Rmd     # must execute before second_notebook.Rmd
#  - functions.R            # file is used by second_notebook.Rmd
#
# Each analysis item is executed to generate md and html files, E.g.:
# 1. <reportdir>/out_md/first_notebook.md
# 2. <reportdir>/out_html/first_notebook.html
analysis: !!omap
- index.Rmd:
- notebooks/nestorowa/import.Rmd:
- notebooks/nestorowa/quality_control.Rmd:
  - notebooks/nestorowa/import.Rmd
- notebooks/nestorowa/normalization.Rmd:
  - notebooks/nestorowa/quality_control.Rmd
- notebooks/nestorowa/further_exploration.Rmd:
  - notebooks/nestorowa/normalization.Rmd
- notebooks/grun/import.Rmd:
- notebooks/grun/quality_control.Rmd:
  - notebooks/grun/import.Rmd
- notebooks/grun/normalization.Rmd:
  - notebooks/grun/quality_control.Rmd
- notebooks/grun/further_exploration.Rmd:
  - notebooks/grun/normalization.Rmd
- notebooks/paul/import.Rmd:
- notebooks/paul/quality_control.Rmd:
  - notebooks/paul/import.Rmd
- notebooks/paul/normalization.Rmd:
  - notebooks/paul/quality_control.Rmd
- notebooks/paul/further_exploration.Rmd:
  - notebooks/paul/normalization.Rmd
- notebooks/merged/merge.Rmd:
  - notebooks/grun/quality_control.Rmd
  - notebooks/paul/quality_control.Rmd
  - notebooks/nestorowa/normalization.Rmd
- notebooks/merged/combined_analysis.Rmd:
  - notebooks/merged/merge.Rmd
version_info:
  snakemake: 6.0.2
  ruamel.yaml: 0.16.12
  scikick: 0.2.1
# Optional site theme customization
output:
  BiocStyle::html_document:
    code_folding: hide
    theme: readable
    toc_float: true
    toc: true
    number_sections: false
    toc_depth: 5
    self_contained: true
```

---

**Functions**


  
  


Next (Project Map)


skmap


cluster\_/

/


cluster\_notebooks/nestorowa/

notebooks/nestorowa/


cluster\_notebooks/grun/

notebooks/grun/


cluster\_notebooks/paul/

notebooks/paul/


cluster\_notebooks/merged/

notebooks/merged/


notebooks/grun/quality\_control.Rmd


Quality Control


notebooks/merged/merge.Rmd


Merge


notebooks/grun/quality\_control.Rmd->notebooks/merged/merge.Rmd


notebooks/grun/normalization.Rmd


Normalization


notebooks/grun/quality\_control.Rmd->notebooks/grun/normalization.Rmd


notebooks/merged/combined\_analysis.Rmd


Combined Analysis


notebooks/merged/merge.Rmd->notebooks/merged/combined\_analysis.Rmd


notebooks/paul/quality\_control.Rmd


Quality Control


notebooks/paul/quality\_control.Rmd->notebooks/merged/merge.Rmd


notebooks/paul/normalization.Rmd


Normalization


notebooks/paul/quality\_control.Rmd->notebooks/paul/normalization.Rmd


notebooks/nestorowa/normalization.Rmd


Normalization


notebooks/nestorowa/normalization.Rmd->notebooks/merged/merge.Rmd


notebooks/nestorowa/further\_exploration.Rmd


Further Exploration


notebooks/nestorowa/normalization.Rmd->notebooks/nestorowa/further\_exploration.Rmd


index.Rmd


Index


notebooks/nestorowa/import.Rmd


Import


notebooks/nestorowa/quality\_control.Rmd


Quality Control


notebooks/nestorowa/import.Rmd->notebooks/nestorowa/quality\_control.Rmd


notebooks/nestorowa/quality\_control.Rmd->notebooks/nestorowa/normalization.Rmd


notebooks/grun/import.Rmd


Import


notebooks/grun/import.Rmd->notebooks/grun/quality\_control.Rmd


notebooks/grun/further\_exploration.Rmd


Further Exploration


notebooks/grun/normalization.Rmd->notebooks/grun/further\_exploration.Rmd


notebooks/paul/import.Rmd


Import


notebooks/paul/import.Rmd->notebooks/paul/quality\_control.Rmd


notebooks/paul/further\_exploration.Rmd


Further Exploration


notebooks/paul/normalization.Rmd->notebooks/paul/further\_exploration.Rmd


---
