# Supplementary material for: Scikick: A sidekick for workflow clarity and reproducibility during extensive data analysis
Source: PLoS One. 2023 Jul 27;18(7):e0289171. doi: 10.1371/journal.pone.0289171 (PMC10374128; doi:10.1371/journal.pone.0289171)
Supplement: S1 File — (ZIP) [file pone.0289171.s001.zip › scikick/docs/scikick_documentation/single-cell_analysis/report/out_html/notebooks/grun/normalization.html]

Normalization and Variance Modelling


Single-cell Analysis

- Nestorowa
  - Import
  - Quality Control
  - Normalization
  - Further Exploration
- Grun
  - Import
  - Quality Control
  - Normalization
  - Further Exploration
- Paul
  - Import
  - Quality Control
  - Normalization
  - Further Exploration
- Merged
  - Merge
  - Combined Analysis

Code 

- Show All Code
- Hide All Code

# Normalization and Variance Modelling

#### 17 February 2023

```
library(scater)
library(scran)
library(BiocStyle)
library(pheatmap)
sce.grun.hsc <- readRDS("output/grun_quality_control_sce.RDS")
```

# Normalization

```
library(scran)
set.seed(101000110)
clusters <- quickCluster(sce.grun.hsc)
sce.grun.hsc <- computeSumFactors(sce.grun.hsc, clusters=clusters)
sce.grun.hsc <- logNormCounts(sce.grun.hsc)
```

We examine some key metrics for the distribution of size factors, and compare it to the library sizes as a sanity check.

```
summary(sizeFactors(sce.grun.hsc))
```

```
    Min.  1st Qu.   Median     Mean  3rd Qu.     Max. 
 0.02746  0.28972  0.60315  1.00000  1.20111 16.43327
```

```
plot(librarySizeFactors(sce.grun.hsc), sizeFactors(sce.grun.hsc), pch=16,
    xlab="Library size factors", ylab="Deconvolution factors", log="xy")
```

Relationship between the library size factors and the deconvolution size factors in the Grun HSC dataset.

# Variance modelling

We create a mean-variance trend based on the expectation that UMI counts have Poisson technical noise.
We do not block on sample here as we want to preserve any difference between the micro-dissected cells and the sorted HSCs.

```
set.seed(00010101)
dec.grun.hsc <- modelGeneVarByPoisson(sce.grun.hsc) 
top.grun.hsc <- getTopHVGs(dec.grun.hsc, prop=0.1)
```

The lack of a typical “bump” shape is caused by the low counts.

```
plot(dec.grun.hsc$mean, dec.grun.hsc$total, pch=16, cex=0.5,
    xlab="Mean of log-expression", ylab="Variance of log-expression")
curfit <- metadata(dec.grun.hsc)
curve(curfit$trend(x), col='dodgerblue', add=TRUE, lwd=2)
```

Per-gene variance as a function of the mean for the log-expression values in the Grun HSC dataset. Each point represents a gene (black) with the mean-variance trend (blue) fitted to the simulated Poisson-distributed noise.

```
saveRDS(sce.grun.hsc,"output/grun_normalization_sce.RDS")
saveRDS(dec.grun.hsc,"output/grun_normalization_dec.RDS")
saveRDS(top.grun.hsc,"output/grun_normalization_top.RDS")
```


---


Click to see page metadata

Computation Started: `2023-02-17 16:45:50`

Finished in `12.451 secs`

---

**Git Log**

No git history available for this page

---

**Packages**

| package | version | date |
| --- | --- | --- |
| MatrixGenerics | 1.2.0 | 2020-10-28 |
| Biobase | 2.50.0 | 2020-10-28 |
| viridis | 0.5.1 | 2020-07-17 |
| edgeR | 3.32.1 | 2021-01-15 |
| BiocSingular | 1.6.0 | 2020-10-28 |
| viridisLite | 0.3.0 | 2020-06-15 |
| DelayedMatrixStats | 1.12.2 | 2021-01-13 |
| scuttle | 1.0.4 | 2020-12-18 |
| assertthat | 0.2.1 | 2020-07-15 |
| statmod | 1.4.35 | 2020-10-20 |
| highr | 0.8 | 2020-07-15 |
| BiocManager | 1.30.10 | 2020-07-15 |
| stats4 | 4.0.1 | 2020-06-07 |
| dqrng | 0.2.1 | 2020-07-15 |
| grDevices | 4.0.1 | 2020-06-07 |
| GenomeInfoDbData | 1.2.4 | 2020-11-03 |
| vipor | 0.4.5 | 2020-07-15 |
| yaml | 2.2.1 | 2020-07-15 |
| pillar | 1.6.0 | 2021-04-14 |
| lattice | 0.20-41 | 2020-06-07 |
| glue | 1.4.2 | 2020-08-28 |
| base | 4.0.1 | 2020-06-07 |
| limma | 3.46.0 | 2020-10-28 |
| beachmat | 2.6.4 | 2020-12-21 |
| digest | 0.6.27 | 2020-10-25 |
| RColorBrewer | 1.1-2 | 2020-07-15 |
| GenomicRanges | 1.42.0 | 2020-10-28 |
| XVector | 0.30.0 | 2020-10-29 |
| colorspace | 2.0-0 | 2020-11-12 |
| htmltools | 0.5.1 | 2021-01-13 |
| Matrix | 1.2-18 | 2020-06-07 |
| pkgconfig | 2.0.3 | 2020-07-15 |
| pheatmap | 1.0.12 | 2020-07-16 |
| zlibbioc | 1.36.0 | 2020-10-29 |
| purrr | 0.3.4 | 2020-07-15 |
| scales | 1.1.1 | 2020-07-16 |
| BiocParallel | 1.24.1 | 2020-11-07 |
| git2r | 0.28.0 | 2021-01-11 |
| tibble | 3.1.1 | 2021-04-19 |
| generics | 0.1.0 | 2020-11-01 |
| datasets | 4.0.1 | 2020-06-07 |
| IRanges | 2.24.1 | 2020-12-13 |
| ggplot2 | 3.3.3 | 2020-12-31 |
| ellipsis | 0.3.1 | 2020-07-15 |
| withr | 2.4.2 | 2021-04-19 |
| SummarizedExperiment | 1.20.0 | 2020-10-28 |
| BiocGenerics | 0.36.0 | 2020-10-28 |
| magrittr | 2.0.1 | 2020-11-18 |
| crayon | 1.4.1 | 2021-02-09 |
| evaluate | 0.14 | 2020-06-15 |
| methods | 4.0.1 | 2020-06-07 |
| fansi | 0.4.2 | 2021-01-16 |
| bluster | 1.0.0 | 2020-10-28 |
| utils | 4.0.1 | 2020-06-07 |
| beeswarm | 0.2.3 | 2020-07-15 |
| tools | 4.0.1 | 2020-06-07 |
| scater | 1.18.3 | 2020-11-09 |
| BiocStyle | 2.18.1 | 2020-11-25 |
| lifecycle | 1.0.0 | 2021-02-16 |
| matrixStats | 0.57.0 | 2020-09-26 |
| stringr | 1.4.0 | 2020-07-15 |
| S4Vectors | 0.28.1 | 2020-12-10 |
| munsell | 0.5.0 | 2020-07-15 |
| locfit | 1.5-9.4 | 2020-07-15 |
| DelayedArray | 0.16.0 | 2020-10-28 |
| irlba | 2.3.3 | 2020-07-15 |
| stats | 4.0.1 | 2020-06-07 |
| compiler | 4.0.1 | 2020-06-07 |
| GenomeInfoDb | 1.26.2 | 2020-12-09 |
| rsvd | 1.0.3 | 2020-07-15 |
| rlang | 0.4.10 | 2020-12-31 |
| grid | 4.0.1 | 2020-06-07 |
| RCurl | 1.98-1.2 | 2020-07-15 |
| BiocNeighbors | 1.8.2 | 2020-12-08 |
| graphics | 4.0.1 | 2020-06-07 |
| SingleCellExperiment | 1.12.0 | 2020-10-28 |
| igraph | 1.2.6 | 2020-10-07 |
| bitops | 1.0-6 | 2020-07-15 |
| rmarkdown | 2.8 | 2021-05-08 |
| gtable | 0.3.0 | 2020-07-15 |
| DBI | 1.1.1 | 2021-01-16 |
| R6 | 2.5.0 | 2020-10-29 |
| gridExtra | 2.3 | 2020-07-15 |
| knitr | 1.30 | 2020-09-23 |
| dplyr | 1.0.5 | 2021-03-06 |
| utf8 | 1.1.4 | 2020-07-15 |
| stringi | 1.5.3 | 2020-09-10 |
| ggbeeswarm | 0.6.0 | 2020-07-16 |
| parallel | 4.0.1 | 2020-06-07 |
| Rcpp | 1.0.6 | 2021-01-16 |
| scran | 1.18.3 | 2020-12-22 |
| vctrs | 0.3.6 | 2020-12-18 |
| tidyselect | 1.1.0 | 2020-07-15 |
| xfun | 0.23 | 2021-05-16 |
| sparseMatrixStats | 1.2.0 | 2020-10-28 |

---

**System Information**

|  | systemInfo |
| --- | --- |
| version | R version 4.0.1 (2020-06-06) |
| platform | x86\_64-apple-darwin17.0 (64-bit) |
| locale | en\_CA.UTF-8 |
| OS | macOS 10.16 |
| UI | X11 |

**Scikick Configuration**

```
cat scikick.yml
```

```
### Scikick Project Workflow Configuration File

# Directory where Scikick will store all standard notebook outputs
reportdir: report

# --- Content below here is best modified by using the Scikick CLI ---

# Notebook Execution Configuration (format summarized below)
# analysis:
#  first_notebook.Rmd:
#  second_notebook.Rmd: 
#  - first_notebook.Rmd     # must execute before second_notebook.Rmd
#  - functions.R            # file is used by second_notebook.Rmd
#
# Each analysis item is executed to generate md and html files, E.g.:
# 1. <reportdir>/out_md/first_notebook.md
# 2. <reportdir>/out_html/first_notebook.html
analysis: !!omap
- index.Rmd:
- notebooks/nestorowa/import.Rmd:
- notebooks/nestorowa/quality_control.Rmd:
  - notebooks/nestorowa/import.Rmd
- notebooks/nestorowa/normalization.Rmd:
  - notebooks/nestorowa/quality_control.Rmd
- notebooks/nestorowa/further_exploration.Rmd:
  - notebooks/nestorowa/normalization.Rmd
- notebooks/grun/import.Rmd:
- notebooks/grun/quality_control.Rmd:
  - notebooks/grun/import.Rmd
- notebooks/grun/normalization.Rmd:
  - notebooks/grun/quality_control.Rmd
- notebooks/grun/further_exploration.Rmd:
  - notebooks/grun/normalization.Rmd
- notebooks/paul/import.Rmd:
- notebooks/paul/quality_control.Rmd:
  - notebooks/paul/import.Rmd
- notebooks/paul/normalization.Rmd:
  - notebooks/paul/quality_control.Rmd
- notebooks/paul/further_exploration.Rmd:
  - notebooks/paul/normalization.Rmd
- notebooks/merged/merge.Rmd:
  - notebooks/grun/quality_control.Rmd
  - notebooks/paul/quality_control.Rmd
  - notebooks/nestorowa/normalization.Rmd
- notebooks/merged/combined_analysis.Rmd:
  - notebooks/merged/merge.Rmd
version_info:
  snakemake: 6.0.2
  ruamel.yaml: 0.16.12
  scikick: 0.2.1
# Optional site theme customization
output:
  BiocStyle::html_document:
    code_folding: hide
    theme: readable
    toc_float: true
    toc: true
    number_sections: false
    toc_depth: 5
    self_contained: true
```

---

**Functions**


  
  


Next (Project Map)


skmap


cluster\_/

/


cluster\_notebooks/nestorowa/

notebooks/nestorowa/


cluster\_notebooks/grun/

notebooks/grun/


cluster\_notebooks/paul/

notebooks/paul/


cluster\_notebooks/merged/

notebooks/merged/


notebooks/grun/quality\_control.Rmd


Quality Control


notebooks/merged/merge.Rmd


Merge


notebooks/grun/quality\_control.Rmd->notebooks/merged/merge.Rmd


notebooks/grun/normalization.Rmd


Normalization


notebooks/grun/quality\_control.Rmd->notebooks/grun/normalization.Rmd


notebooks/merged/combined\_analysis.Rmd


Combined Analysis


notebooks/merged/merge.Rmd->notebooks/merged/combined\_analysis.Rmd


notebooks/paul/quality\_control.Rmd


Quality Control


notebooks/paul/quality\_control.Rmd->notebooks/merged/merge.Rmd


notebooks/paul/normalization.Rmd


Normalization


notebooks/paul/quality\_control.Rmd->notebooks/paul/normalization.Rmd


notebooks/nestorowa/normalization.Rmd


Normalization


notebooks/nestorowa/normalization.Rmd->notebooks/merged/merge.Rmd


notebooks/nestorowa/further\_exploration.Rmd


Further Exploration


notebooks/nestorowa/normalization.Rmd->notebooks/nestorowa/further\_exploration.Rmd


index.Rmd


Index


notebooks/nestorowa/import.Rmd


Import


notebooks/nestorowa/quality\_control.Rmd


Quality Control


notebooks/nestorowa/import.Rmd->notebooks/nestorowa/quality\_control.Rmd


notebooks/nestorowa/quality\_control.Rmd->notebooks/nestorowa/normalization.Rmd


notebooks/grun/import.Rmd


Import


notebooks/grun/import.Rmd->notebooks/grun/quality\_control.Rmd


notebooks/grun/further\_exploration.Rmd


Further Exploration


notebooks/grun/normalization.Rmd->notebooks/grun/further\_exploration.Rmd


notebooks/paul/import.Rmd


Import


notebooks/paul/import.Rmd->notebooks/paul/quality\_control.Rmd


notebooks/paul/further\_exploration.Rmd


Further Exploration


notebooks/paul/normalization.Rmd->notebooks/paul/further\_exploration.Rmd


---
