# Supplementary material for: Scikick: A sidekick for workflow clarity and reproducibility during extensive data analysis
Source: PLoS One. 2023 Jul 27;18(7):e0289171. doi: 10.1371/journal.pone.0289171 (PMC10374128; doi:10.1371/journal.pone.0289171)
Supplement: S1 File — (ZIP) [file pone.0289171.s001.zip › scikick/docs/scikick_documentation/single-cell_analysis/report/out_html/notebooks/nestorowa/quality_control.html]

Quality Control


Single-cell Analysis

- Nestorowa
  - Import
  - Quality Control
  - Normalization
  - Further Exploration
- Grun
  - Import
  - Quality Control
  - Normalization
  - Further Exploration
- Paul
  - Import
  - Quality Control
  - Normalization
  - Further Exploration
- Merged
  - Merge
  - Combined Analysis

Code 

- Show All Code
- Hide All Code

# Quality Control

#### 17 February 2023

```
sce.nest <- readRDS("output/nestorowa_import_sce.RDS")
```

```
unfiltered <- sce.nest
```

For some reason, no mitochondrial transcripts are available, so we will perform quality control using the spike-in proportions only.

```
library(scater)
stats <- perCellQCMetrics(sce.nest)
qc <- quickPerCellQC(stats, percent_subsets="altexps_ERCC_percent")
sce.nest <- sce.nest[,!qc$discard]
```

We examine the number of cells discarded for each reason.

```
colSums(as.matrix(qc))
```

```
             low_lib_size            low_n_features high_altexps_ERCC_percent                   discard 
                      146                        28                       241                       264
```

We create some diagnostic plots for each metric.

```
colData(unfiltered) <- cbind(colData(unfiltered), stats)
unfiltered$discard <- qc$discard

gridExtra::grid.arrange(
    plotColData(unfiltered, y="sum", colour_by="discard") +
        scale_y_log10() + ggtitle("Total count"),
    plotColData(unfiltered, y="detected", colour_by="discard") +
        scale_y_log10() + ggtitle("Detected features"),
    plotColData(unfiltered, y="altexps_ERCC_percent",
        colour_by="discard") + ggtitle("ERCC percent"),
    ncol=2
)
```

Distribution of each QC metric across cells in the Nestorowa HSC dataset. Each point represents a cell and is colored according to whether that cell was discarded.

```
saveRDS(sce.nest,"output/nestorowa_quality_control_sce.RDS")
```


---


Click to see page metadata

Computation Started: `2023-02-17 16:43:00`

Finished in `23.85 secs`

---

**Git Log**

No git history available for this page

---

**Packages**

| package | version | date |
| --- | --- | --- |
| Rcpp | 1.0.6 | 2021-01-16 |
| rsvd | 1.0.3 | 2020-07-15 |
| lattice | 0.20-41 | 2020-06-07 |
| digest | 0.6.27 | 2020-10-25 |
| assertthat | 0.2.1 | 2020-07-15 |
| SingleCellExperiment | 1.12.0 | 2020-10-28 |
| utf8 | 1.1.4 | 2020-07-15 |
| R6 | 2.5.0 | 2020-10-29 |
| GenomeInfoDb | 1.26.2 | 2020-12-09 |
| stats4 | 4.0.1 | 2020-06-07 |
| evaluate | 0.14 | 2020-06-15 |
| highr | 0.8 | 2020-07-15 |
| ggplot2 | 3.3.3 | 2020-12-31 |
| pillar | 1.6.0 | 2021-04-14 |
| sparseMatrixStats | 1.2.0 | 2020-10-28 |
| utils | 4.0.1 | 2020-06-07 |
| zlibbioc | 1.36.0 | 2020-10-29 |
| rlang | 0.4.10 | 2020-12-31 |
| irlba | 2.3.3 | 2020-07-15 |
| S4Vectors | 0.28.1 | 2020-12-10 |
| Matrix | 1.2-18 | 2020-06-07 |
| labeling | 0.4.2 | 2020-10-21 |
| BiocNeighbors | 1.8.2 | 2020-12-08 |
| BiocParallel | 1.24.1 | 2020-11-07 |
| stringr | 1.4.0 | 2020-07-15 |
| RCurl | 1.98-1.2 | 2020-07-15 |
| munsell | 0.5.0 | 2020-07-15 |
| beachmat | 2.6.4 | 2020-12-21 |
| DelayedArray | 0.16.0 | 2020-10-28 |
| compiler | 4.0.1 | 2020-06-07 |
| vipor | 0.4.5 | 2020-07-15 |
| BiocSingular | 1.6.0 | 2020-10-28 |
| xfun | 0.23 | 2021-05-16 |
| pkgconfig | 2.0.3 | 2020-07-15 |
| stats | 4.0.1 | 2020-06-07 |
| BiocGenerics | 0.36.0 | 2020-10-28 |
| ggbeeswarm | 0.6.0 | 2020-07-16 |
| tidyselect | 1.1.0 | 2020-07-15 |
| SummarizedExperiment | 1.20.0 | 2020-10-28 |
| tibble | 3.1.1 | 2021-04-19 |
| gridExtra | 2.3 | 2020-07-15 |
| GenomeInfoDbData | 1.2.4 | 2020-11-03 |
| IRanges | 2.24.1 | 2020-12-13 |
| matrixStats | 0.57.0 | 2020-09-26 |
| grDevices | 4.0.1 | 2020-06-07 |
| viridisLite | 0.3.0 | 2020-06-15 |
| fansi | 0.4.2 | 2021-01-16 |
| crayon | 1.4.1 | 2021-02-09 |
| dplyr | 1.0.5 | 2021-03-06 |
| withr | 2.4.2 | 2021-04-19 |
| bitops | 1.0-6 | 2020-07-15 |
| grid | 4.0.1 | 2020-06-07 |
| gtable | 0.3.0 | 2020-07-15 |
| lifecycle | 1.0.0 | 2021-02-16 |
| DBI | 1.1.1 | 2021-01-16 |
| git2r | 0.28.0 | 2021-01-11 |
| magrittr | 2.0.1 | 2020-11-18 |
| datasets | 4.0.1 | 2020-06-07 |
| scales | 1.1.1 | 2020-07-16 |
| stringi | 1.5.3 | 2020-09-10 |
| scuttle | 1.0.4 | 2020-12-18 |
| farver | 2.0.3 | 2020-07-15 |
| XVector | 0.30.0 | 2020-10-29 |
| viridis | 0.5.1 | 2020-07-17 |
| scater | 1.18.3 | 2020-11-09 |
| DelayedMatrixStats | 1.12.2 | 2021-01-13 |
| ellipsis | 0.3.1 | 2020-07-15 |
| graphics | 4.0.1 | 2020-06-07 |
| generics | 0.1.0 | 2020-11-01 |
| vctrs | 0.3.6 | 2020-12-18 |
| cowplot | 1.1.1 | 2020-12-31 |
| base | 4.0.1 | 2020-06-07 |
| tools | 4.0.1 | 2020-06-07 |
| Biobase | 2.50.0 | 2020-10-28 |
| glue | 1.4.2 | 2020-08-28 |
| beeswarm | 0.2.3 | 2020-07-15 |
| purrr | 0.3.4 | 2020-07-15 |
| MatrixGenerics | 1.2.0 | 2020-10-28 |
| parallel | 4.0.1 | 2020-06-07 |
| colorspace | 2.0-0 | 2020-11-12 |
| GenomicRanges | 1.42.0 | 2020-10-28 |
| knitr | 1.30 | 2020-09-23 |
| methods | 4.0.1 | 2020-06-07 |

---

**System Information**

|  | systemInfo |
| --- | --- |
| version | R version 4.0.1 (2020-06-06) |
| platform | x86\_64-apple-darwin17.0 (64-bit) |
| locale | en\_CA.UTF-8 |
| OS | macOS 10.16 |
| UI | X11 |

**Scikick Configuration**

```
cat scikick.yml
```

```
### Scikick Project Workflow Configuration File

# Directory where Scikick will store all standard notebook outputs
reportdir: report

# --- Content below here is best modified by using the Scikick CLI ---

# Notebook Execution Configuration (format summarized below)
# analysis:
#  first_notebook.Rmd:
#  second_notebook.Rmd: 
#  - first_notebook.Rmd     # must execute before second_notebook.Rmd
#  - functions.R            # file is used by second_notebook.Rmd
#
# Each analysis item is executed to generate md and html files, E.g.:
# 1. <reportdir>/out_md/first_notebook.md
# 2. <reportdir>/out_html/first_notebook.html
analysis: !!omap
- index.Rmd:
- notebooks/import.Rmd:
- notebooks/quality_control.Rmd:
  - notebooks/import.Rmd
- notebooks/normalization.Rmd:
  - notebooks/quality_control.Rmd
- notebooks/further_exploration.Rmd:
  - notebooks/normalization.Rmd
version_info:
  snakemake: 6.0.2
  ruamel.yaml: 0.16.12
  scikick: 0.2.1
# Optional site theme customization
output:
  BiocStyle::html_document:
    code_folding: hide
    theme: readable
    toc_float: true
    toc: true
    number_sections: false
    toc_depth: 5
    self_contained: true
```

---

**Functions**


  
  


Next (Project Map)


skmap


cluster\_notebooks/grun/

notebooks/grun/


cluster\_notebooks/paul/

notebooks/paul/


cluster\_/

/


cluster\_notebooks/nestorowa/

notebooks/nestorowa/


cluster\_notebooks/merged/

notebooks/merged/


notebooks/grun/quality\_control.Rmd


Quality Control


notebooks/merged/merge.Rmd


Merge


notebooks/grun/quality\_control.Rmd->notebooks/merged/merge.Rmd


notebooks/grun/normalization.Rmd


Normalization


notebooks/grun/quality\_control.Rmd->notebooks/grun/normalization.Rmd


notebooks/merged/combined\_analysis.Rmd


Combined Analysis


notebooks/merged/merge.Rmd->notebooks/merged/combined\_analysis.Rmd


notebooks/paul/quality\_control.Rmd


Quality Control


notebooks/paul/quality\_control.Rmd->notebooks/merged/merge.Rmd


notebooks/paul/normalization.Rmd


Normalization


notebooks/paul/quality\_control.Rmd->notebooks/paul/normalization.Rmd


notebooks/nestorowa/normalization.Rmd


Normalization


notebooks/nestorowa/normalization.Rmd->notebooks/merged/merge.Rmd


notebooks/nestorowa/further\_exploration.Rmd


Further Exploration


notebooks/nestorowa/normalization.Rmd->notebooks/nestorowa/further\_exploration.Rmd


index.Rmd


Index


notebooks/nestorowa/import.Rmd


Import


notebooks/nestorowa/quality\_control.Rmd


Quality Control


notebooks/nestorowa/import.Rmd->notebooks/nestorowa/quality\_control.Rmd


notebooks/nestorowa/quality\_control.Rmd->notebooks/nestorowa/normalization.Rmd


notebooks/grun/import.Rmd


Import


notebooks/grun/import.Rmd->notebooks/grun/quality\_control.Rmd


notebooks/grun/further\_exploration.Rmd


Further Exploration


notebooks/grun/normalization.Rmd->notebooks/grun/further\_exploration.Rmd


notebooks/paul/import.Rmd


Import


notebooks/paul/import.Rmd->notebooks/paul/quality\_control.Rmd


notebooks/paul/further\_exploration.Rmd


Further Exploration


notebooks/paul/normalization.Rmd->notebooks/paul/further\_exploration.Rmd


---
