# Supplementary material for: Scikick: A sidekick for workflow clarity and reproducibility during extensive data analysis
Source: PLoS One. 2023 Jul 27;18(7):e0289171. doi: 10.1371/journal.pone.0289171 (PMC10374128; doi:10.1371/journal.pone.0289171)
Supplement: S1 File — (ZIP) [file pone.0289171.s001.zip › scikick/docs/scikick_documentation/single-cell_analysis/report/out_html/notebooks/merged/merge.html]

Merge


Single-cell Analysis

- Nestorowa
  - Import
  - Quality Control
  - Normalization
  - Further Exploration
- Grun
  - Import
  - Quality Control
  - Normalization
  - Further Exploration
- Paul
  - Import
  - Quality Control
  - Normalization
  - Further Exploration
- Merged
  - Merge
  - Combined Analysis

Code 

- Show All Code
- Hide All Code

# Merge

#### 17 February 2023

# Introduction

The blood is probably the most well-studied tissue in the single-cell field, mostly because everything is already dissociated “for free”.
Of particular interest has been the use of single-cell genomics to study cell fate decisions in haematopoeisis.
Indeed, it was not long ago that dueling interpretations of haematopoeitic stem cell (HSC) datasets were a mainstay of single-cell conferences.
Sadly, these times have mostly passed so we will instead entertain ourselves by combining a small number of these datasets into a single analysis.

# Data loading

```
sce.nest
```

```
class: SingleCellExperiment 
dim: 46078 1656 
metadata(0):
assays(2): counts logcounts
rownames(46078): ENSMUSG00000000001 ENSMUSG00000000003 ... ENSMUSG00000107391
  ENSMUSG00000107392
rowData names(3): GENEID SYMBOL SEQNAME
colnames(1656): HSPC_025 HSPC_031 ... Prog_852 Prog_810
colData names(3): cell.type FACS sizeFactor
reducedDimNames(1): diffusion
altExpNames(1): ERCC
```

The Grun dataset requires a little bit of subsetting and re-analysis to only consider the sorted HSCs.

```
library(scuttle)
sce.grun.hsc <- sce.grun.hsc[,sce.grun.hsc$protocol=="sorted hematopoietic stem cells"]
sce.grun.hsc <- logNormCounts(sce.grun.hsc)

set.seed(11001)
library(scran)
dec.grun.hsc <- modelGeneVarByPoisson(sce.grun.hsc)
```

Finally, we will grab the Paul dataset, which we will also subset to only consider the unsorted myeloid population.
This removes the various knockout conditions that just complicates matters.

```
sce.paul <- sce.paul[,sce.paul$Batch_desc=="Unsorted myeloid"]
sce.paul <- logNormCounts(sce.paul)

set.seed(00010010)
dec.paul <- modelGeneVarByPoisson(sce.paul)
```

# Setting up the merge

```
common <- Reduce(intersect, list(rownames(sce.nest),
    rownames(sce.grun.hsc), rownames(sce.paul)))
length(common)
```

```
[1] 17147
```

Combining variances to obtain a single set of HVGs.

```
combined.dec <- combineVar(
    dec.nest[common,], 
    dec.grun.hsc[common,], 
    dec.paul[common,]
)
hvgs <- getTopHVGs(combined.dec, n=5000)
```

Adjusting for gross differences in sequencing depth.

```
library(batchelor)
normed.sce <- multiBatchNorm(
    Nestorowa=sce.nest[common,],
    Grun=sce.grun.hsc[common,],
    Paul=sce.paul[common,]
)
```

# Merging the datasets

We turn on `auto.merge=TRUE` to instruct `fastMNN()` to merge the batch that offers the largest number of MNNs.
This aims to perform the “easiest” merges first, i.e., between the most replicate-like batches,
before tackling merges between batches that have greater differences in their population composition.

```
set.seed(1000010)
merged <- fastMNN(normed.sce, subset.row=hvgs, auto.merge=TRUE)
```

Not too much variance lost inside each batch, hopefully.
We also observe that the algorithm chose to merge the more diverse Nestorowa and Paul datasets before dealing with the HSC-only Grun dataset.

```
metadata(merged)$merge.info[,c("left", "right", "lost.var")]
```

```
DataFrame with 2 rows and 3 columns
            left     right                        lost.var
          <List>    <List>                        <matrix>
1           Paul Nestorowa 0.01069374:0.0000000:0.00739465
2 Paul,Nestorowa      Grun 0.00562344:0.0178334:0.00702615
```

```
saveRDS(merged,"output/merged_sce.RDS")
```


---


Click to see page metadata

Computation Started: `2023-02-17 16:46:04`

Finished in `26.822 secs`

---

**Git Log**

No git history available for this page

---

**Packages**

| package | version | date |
| --- | --- | --- |
| Rcpp | 1.0.6 | 2021-01-16 |
| git2r | 0.28.0 | 2021-01-11 |
| batchelor | 1.6.2 | 2020-11-27 |
| compiler | 4.0.1 | 2020-06-07 |
| bluster | 1.0.0 | 2020-10-28 |
| GenomeInfoDb | 1.26.2 | 2020-12-09 |
| XVector | 0.30.0 | 2020-10-29 |
| MatrixGenerics | 1.2.0 | 2020-10-28 |
| methods | 4.0.1 | 2020-06-07 |
| bitops | 1.0-6 | 2020-07-15 |
| BiocNeighbors | 1.8.2 | 2020-12-08 |
| utils | 4.0.1 | 2020-06-07 |
| tools | 4.0.1 | 2020-06-07 |
| DelayedMatrixStats | 1.12.2 | 2021-01-13 |
| grDevices | 4.0.1 | 2020-06-07 |
| zlibbioc | 1.36.0 | 2020-10-29 |
| statmod | 1.4.35 | 2020-10-20 |
| SingleCellExperiment | 1.12.0 | 2020-10-28 |
| evaluate | 0.14 | 2020-06-15 |
| lattice | 0.20-41 | 2020-06-07 |
| pkgconfig | 2.0.3 | 2020-07-15 |
| Matrix | 1.2-18 | 2020-06-07 |
| igraph | 1.2.6 | 2020-10-07 |
| DelayedArray | 0.16.0 | 2020-10-28 |
| parallel | 4.0.1 | 2020-06-07 |
| xfun | 0.23 | 2021-05-16 |
| GenomeInfoDbData | 1.2.4 | 2020-11-03 |
| stringr | 1.4.0 | 2020-07-15 |
| knitr | 1.30 | 2020-09-23 |
| S4Vectors | 0.28.1 | 2020-12-10 |
| graphics | 4.0.1 | 2020-06-07 |
| datasets | 4.0.1 | 2020-06-07 |
| stats | 4.0.1 | 2020-06-07 |
| IRanges | 2.24.1 | 2020-12-13 |
| stats4 | 4.0.1 | 2020-06-07 |
| locfit | 1.5-9.4 | 2020-07-15 |
| grid | 4.0.1 | 2020-06-07 |
| scuttle | 1.0.4 | 2020-12-18 |
| base | 4.0.1 | 2020-06-07 |
| Biobase | 2.50.0 | 2020-10-28 |
| BiocParallel | 1.24.1 | 2020-11-07 |
| limma | 3.46.0 | 2020-10-28 |
| irlba | 2.3.3 | 2020-07-15 |
| magrittr | 2.0.1 | 2020-11-18 |
| BiocSingular | 1.6.0 | 2020-10-28 |
| edgeR | 3.32.1 | 2021-01-15 |
| matrixStats | 0.57.0 | 2020-09-26 |
| sparseMatrixStats | 1.2.0 | 2020-10-28 |
| BiocGenerics | 0.36.0 | 2020-10-28 |
| GenomicRanges | 1.42.0 | 2020-10-28 |
| beachmat | 2.6.4 | 2020-12-21 |
| SummarizedExperiment | 1.20.0 | 2020-10-28 |
| rsvd | 1.0.3 | 2020-07-15 |
| dqrng | 0.2.1 | 2020-07-15 |
| ResidualMatrix | 1.0.0 | 2020-10-28 |
| stringi | 1.5.3 | 2020-09-10 |
| RCurl | 1.98-1.2 | 2020-07-15 |
| scran | 1.18.3 | 2020-12-22 |

---

**System Information**

|  | systemInfo |
| --- | --- |
| version | R version 4.0.1 (2020-06-06) |
| platform | x86\_64-apple-darwin17.0 (64-bit) |
| locale | en\_CA.UTF-8 |
| OS | macOS 10.16 |
| UI | X11 |

**Scikick Configuration**

```
cat scikick.yml
```

```
### Scikick Project Workflow Configuration File

# Directory where Scikick will store all standard notebook outputs
reportdir: report

# --- Content below here is best modified by using the Scikick CLI ---

# Notebook Execution Configuration (format summarized below)
# analysis:
#  first_notebook.Rmd:
#  second_notebook.Rmd: 
#  - first_notebook.Rmd     # must execute before second_notebook.Rmd
#  - functions.R            # file is used by second_notebook.Rmd
#
# Each analysis item is executed to generate md and html files, E.g.:
# 1. <reportdir>/out_md/first_notebook.md
# 2. <reportdir>/out_html/first_notebook.html
analysis: !!omap
- index.Rmd:
- notebooks/nestorowa/import.Rmd:
- notebooks/nestorowa/quality_control.Rmd:
  - notebooks/nestorowa/import.Rmd
- notebooks/nestorowa/normalization.Rmd:
  - notebooks/nestorowa/quality_control.Rmd
- notebooks/nestorowa/further_exploration.Rmd:
  - notebooks/nestorowa/normalization.Rmd
- notebooks/grun/import.Rmd:
- notebooks/grun/quality_control.Rmd:
  - notebooks/grun/import.Rmd
- notebooks/grun/normalization.Rmd:
  - notebooks/grun/quality_control.Rmd
- notebooks/grun/further_exploration.Rmd:
  - notebooks/grun/normalization.Rmd
- notebooks/paul/import.Rmd:
- notebooks/paul/quality_control.Rmd:
  - notebooks/paul/import.Rmd
- notebooks/paul/normalization.Rmd:
  - notebooks/paul/quality_control.Rmd
- notebooks/paul/further_exploration.Rmd:
  - notebooks/paul/normalization.Rmd
- notebooks/merged/merge.Rmd:
  - notebooks/grun/quality_control.Rmd
  - notebooks/paul/quality_control.Rmd
  - notebooks/nestorowa/normalization.Rmd
- notebooks/merged/combined_analysis.Rmd:
  - notebooks/merged/merge.Rmd
version_info:
  snakemake: 6.0.2
  ruamel.yaml: 0.16.12
  scikick: 0.2.1
# Optional site theme customization
output:
  BiocStyle::html_document:
    code_folding: hide
    theme: readable
    toc_float: true
    toc: true
    number_sections: false
    toc_depth: 5
    self_contained: true
```

---

**Functions**


  
  


Next (Project Map)


skmap


cluster\_notebooks/merged/

notebooks/merged/


cluster\_/

/


cluster\_notebooks/nestorowa/

notebooks/nestorowa/


cluster\_notebooks/grun/

notebooks/grun/


cluster\_notebooks/paul/

notebooks/paul/


notebooks/grun/quality\_control.Rmd


Quality Control


notebooks/merged/merge.Rmd


Merge


notebooks/grun/quality\_control.Rmd->notebooks/merged/merge.Rmd


notebooks/grun/normalization.Rmd


Normalization


notebooks/grun/quality\_control.Rmd->notebooks/grun/normalization.Rmd


notebooks/merged/combined\_analysis.Rmd


Combined Analysis


notebooks/merged/merge.Rmd->notebooks/merged/combined\_analysis.Rmd


notebooks/paul/quality\_control.Rmd


Quality Control


notebooks/paul/quality\_control.Rmd->notebooks/merged/merge.Rmd


notebooks/paul/normalization.Rmd


Normalization


notebooks/paul/quality\_control.Rmd->notebooks/paul/normalization.Rmd


notebooks/nestorowa/normalization.Rmd


Normalization


notebooks/nestorowa/normalization.Rmd->notebooks/merged/merge.Rmd


notebooks/nestorowa/further\_exploration.Rmd


Further Exploration


notebooks/nestorowa/normalization.Rmd->notebooks/nestorowa/further\_exploration.Rmd


index.Rmd


Index


notebooks/nestorowa/import.Rmd


Import


notebooks/nestorowa/quality\_control.Rmd


Quality Control


notebooks/nestorowa/import.Rmd->notebooks/nestorowa/quality\_control.Rmd


notebooks/nestorowa/quality\_control.Rmd->notebooks/nestorowa/normalization.Rmd


notebooks/grun/import.Rmd


Import


notebooks/grun/import.Rmd->notebooks/grun/quality\_control.Rmd


notebooks/grun/further\_exploration.Rmd


Further Exploration


notebooks/grun/normalization.Rmd->notebooks/grun/further\_exploration.Rmd


notebooks/paul/import.Rmd


Import


notebooks/paul/import.Rmd->notebooks/paul/quality\_control.Rmd


notebooks/paul/further\_exploration.Rmd


Further Exploration


notebooks/paul/normalization.Rmd->notebooks/paul/further\_exploration.Rmd


---
