# Supplementary material for: Scikick: A sidekick for workflow clarity and reproducibility during extensive data analysis
Source: PLoS One. 2023 Jul 27;18(7):e0289171. doi: 10.1371/journal.pone.0289171 (PMC10374128; doi:10.1371/journal.pone.0289171)
Supplement: S1 File — (ZIP) [file pone.0289171.s001.zip › scikick/docs/scikick_documentation/single-cell_analysis/report/out_html/notebooks/merged/combined_analysis.html]

Combined analyses


Single-cell Analysis

- Nestorowa
  - Import
  - Quality Control
  - Normalization
  - Further Exploration
- Grun
  - Import
  - Quality Control
  - Normalization
  - Further Exploration
- Paul
  - Import
  - Quality Control
  - Normalization
  - Further Exploration
- Merged
  - Merge
  - Combined Analysis

Code 

- Show All Code
- Hide All Code

# Combined analyses

#### 17 February 2023

```
library(scater)
library(scran)
library(BiocStyle)
library(pheatmap)
merged <- readRDS("output/merged_sce.RDS")
```

The Grun dataset does not contribute to many clusters, consistent with a pure undifferentiated HSC population.
Most of the other clusters contain contributions from the Nestorowa and Paul datasets, though some are unique to the Paul dataset.
This may be due to incomplete correction though we tend to think that this are Paul-specific subpopulations,
given that the Nestorowa dataset does not have similarly sized unique clusters that might represent their uncorrected counterparts.

```
library(bluster)
colLabels(merged) <- clusterRows(reducedDim(merged), 
    NNGraphParam(cluster.fun="louvain"))
table(Cluster=colLabels(merged), Batch=merged$batch)
```

```
       Batch
Cluster Grun Nestorowa Paul
     1     0        40  206
     2     0        19    0
     3    39       353  146
     4     0         6   29
     5     0       217  487
     6     0       162  522
     7     0       133  191
     8    22       411   94
     9   230       315  348
     10    0         0  385
     11    0         0  397
```

While I prefer \(t\)-SNE plots,
we’ll switch to a UMAP plot to highlight some of the trajectory-like structure across clusters.

```
library(scater)
set.seed(101010101)
merged <- runUMAP(merged, dimred="corrected")
gridExtra::grid.arrange(
    plotUMAP(merged, colour_by="label"),
    plotUMAP(merged, colour_by="batch"),
    ncol=2
)
```

Obligatory UMAP plot of the merged HSC datasets, where each point represents a cell and is colored by the batch of origin (left) or its assigned cluster (right).

In fact, we might as well compute a trajectory right now.
*TSCAN* constructs a reasonable minimum spanning tree but the path choices are somewhat incongruent with the UMAP coordinates.
This is most likely due to the fact that *TSCAN* operates on cluster centroids,
which is simple and efficient but does not consider the variance of cells within each cluster.
It is entirely possible for two well-separated clusters to be closer than two adjacent clusters if the latter span a wider region of the coordinate space.

```
library(TSCAN)
pseudo.out <- quickPseudotime(merged, use.dimred="corrected", outgroup=TRUE)
```

```
common.pseudo <- rowMeans(pseudo.out$ordering, na.rm=TRUE)
plotUMAP(merged, colour_by=I(common.pseudo), 
        text_by="label", text_colour="red") +
    geom_line(data=pseudo.out$connected$UMAP, 
        mapping=aes(x=dim1, y=dim2, group=edge))
```

Another UMAP plot of the merged HSC datasets, where each point represents a cell and is colored by its *TSCAN* pseudotime. The lines correspond to the edges of the MST across cluster centers.

To fix this, we construct the minimum spanning tree using distances based on pairs of mutual nearest neighbors between clusters.
This focuses on the closeness of the boundaries of each pair of clusters rather than their centroids,
ensuring that adjacent clusters are connected even if their centroids are far apart.
Doing so yields a trajectory that is more consistent with the visual connections on the UMAP plot.

```
pseudo.out2 <- quickPseudotime(merged, use.dimred="corrected", 
    with.mnn=TRUE, outgroup=TRUE)

common.pseudo2 <- rowMeans(pseudo.out2$ordering, na.rm=TRUE)
plotUMAP(merged, colour_by=I(common.pseudo2), 
        text_by="label", text_colour="red") +
    geom_line(data=pseudo.out2$connected$UMAP, 
        mapping=aes(x=dim1, y=dim2, group=edge))
```

Yet another UMAP plot of the merged HSC datasets, where each point represents a cell and is colored by its *TSCAN* pseudotime. The lines correspond to the edges of the MST across cluster centers.


---


Click to see page metadata

Computation Started: `2023-02-17 16:46:31`

Finished in `18.783 secs`

---

**Git Log**

No git history available for this page

---

**Packages**

| package | version | date |
| --- | --- | --- |
| nlme | 3.1-148 | 2020-06-07 |
| TSCAN | 1.28.0 | 2020-10-28 |
| utils | 4.0.1 | 2020-06-07 |
| bitops | 1.0-6 | 2020-07-15 |
| matrixStats | 0.57.0 | 2020-09-26 |
| RcppAnnoy | 0.0.18 | 2020-12-16 |
| RColorBrewer | 1.1-2 | 2020-07-15 |
| GenomeInfoDb | 1.26.2 | 2020-12-09 |
| tools | 4.0.1 | 2020-06-07 |
| ResidualMatrix | 1.0.0 | 2020-10-28 |
| utf8 | 1.1.4 | 2020-07-15 |
| R6 | 2.5.0 | 2020-10-29 |
| irlba | 2.3.3 | 2020-07-15 |
| KernSmooth | 2.23-17 | 2020-06-07 |
| vipor | 0.4.5 | 2020-07-15 |
| mgcv | 1.8-31 | 2020-06-07 |
| uwot | 0.1.10 | 2020-12-16 |
| DBI | 1.1.1 | 2021-01-16 |
| BiocGenerics | 0.36.0 | 2020-10-28 |
| colorspace | 2.0-0 | 2020-11-12 |
| withr | 2.4.2 | 2021-04-19 |
| tidyselect | 1.1.0 | 2020-07-15 |
| gridExtra | 2.3 | 2020-07-15 |
| git2r | 0.28.0 | 2021-01-11 |
| compiler | 4.0.1 | 2020-06-07 |
| Biobase | 2.50.0 | 2020-10-28 |
| BiocNeighbors | 1.8.2 | 2020-12-08 |
| datasets | 4.0.1 | 2020-06-07 |
| DelayedArray | 0.16.0 | 2020-10-28 |
| labeling | 0.4.2 | 2020-10-21 |
| base | 4.0.1 | 2020-06-07 |
| caTools | 1.18.1 | 2021-01-12 |
| scales | 1.1.1 | 2020-07-16 |
| stringr | 1.4.0 | 2020-07-15 |
| digest | 0.6.27 | 2020-10-25 |
| rmarkdown | 2.8 | 2021-05-08 |
| XVector | 0.30.0 | 2020-10-29 |
| scater | 1.18.3 | 2020-11-09 |
| pkgconfig | 2.0.3 | 2020-07-15 |
| htmltools | 0.5.1 | 2021-01-13 |
| sparseMatrixStats | 1.2.0 | 2020-10-28 |
| MatrixGenerics | 1.2.0 | 2020-10-28 |
| fastmap | 1.0.1 | 2020-07-15 |
| limma | 3.46.0 | 2020-10-28 |
| grDevices | 4.0.1 | 2020-06-07 |
| highr | 0.8 | 2020-07-15 |
| rlang | 0.4.10 | 2020-12-31 |
| shiny | 1.5.0 | 2020-07-16 |
| DelayedMatrixStats | 1.12.2 | 2021-01-13 |
| generics | 0.1.0 | 2020-11-01 |
| farver | 2.0.3 | 2020-07-15 |
| combinat | 0.0-8 | 2020-07-15 |
| mclust | 5.4.7 | 2020-11-21 |
| gtools | 3.8.2 | 2020-07-15 |
| BiocParallel | 1.24.1 | 2020-11-07 |
| dplyr | 1.0.5 | 2021-03-06 |
| RCurl | 1.98-1.2 | 2020-07-15 |
| magrittr | 2.0.1 | 2020-11-18 |
| BiocSingular | 1.6.0 | 2020-10-28 |
| GenomeInfoDbData | 1.2.4 | 2020-11-03 |
| scuttle | 1.0.4 | 2020-12-18 |
| Matrix | 1.2-18 | 2020-06-07 |
| Rcpp | 1.0.6 | 2021-01-16 |
| ggbeeswarm | 0.6.0 | 2020-07-16 |
| munsell | 0.5.0 | 2020-07-15 |
| S4Vectors | 0.28.1 | 2020-12-10 |
| fansi | 0.4.2 | 2021-01-16 |
| viridis | 0.5.1 | 2020-07-17 |
| lifecycle | 1.0.0 | 2021-02-16 |
| stringi | 1.5.3 | 2020-09-10 |
| yaml | 2.2.1 | 2020-07-15 |
| edgeR | 3.32.1 | 2021-01-15 |
| SummarizedExperiment | 1.20.0 | 2020-10-28 |
| zlibbioc | 1.36.0 | 2020-10-29 |
| plyr | 1.8.6 | 2020-07-15 |
| gplots | 3.1.1 | 2020-11-29 |
| grid | 4.0.1 | 2020-06-07 |
| promises | 1.1.1 | 2020-07-16 |
| parallel | 4.0.1 | 2020-06-07 |
| dqrng | 0.2.1 | 2020-07-15 |
| crayon | 1.4.1 | 2021-02-09 |
| methods | 4.0.1 | 2020-06-07 |
| lattice | 0.20-41 | 2020-06-07 |
| splines | 4.0.1 | 2020-06-07 |
| cowplot | 1.1.1 | 2020-12-31 |
| beachmat | 2.6.4 | 2020-12-21 |
| locfit | 1.5-9.4 | 2020-07-15 |
| knitr | 1.30 | 2020-09-23 |
| pillar | 1.6.0 | 2021-04-14 |
| igraph | 1.2.6 | 2020-10-07 |
| GenomicRanges | 1.42.0 | 2020-10-28 |
| codetools | 0.2-16 | 2020-06-07 |
| stats4 | 4.0.1 | 2020-06-07 |
| glue | 1.4.2 | 2020-08-28 |
| evaluate | 0.14 | 2020-06-15 |
| scran | 1.18.3 | 2020-12-22 |
| BiocManager | 1.30.10 | 2020-07-15 |
| batchelor | 1.6.2 | 2020-11-27 |
| httpuv | 1.5.5 | 2021-01-13 |
| vctrs | 0.3.6 | 2020-12-18 |
| graphics | 4.0.1 | 2020-06-07 |
| gtable | 0.3.0 | 2020-07-15 |
| purrr | 0.3.4 | 2020-07-15 |
| assertthat | 0.2.1 | 2020-07-15 |
| ggplot2 | 3.3.3 | 2020-12-31 |
| xfun | 0.23 | 2021-05-16 |
| mime | 0.9 | 2020-07-15 |
| rsvd | 1.0.3 | 2020-07-15 |
| xtable | 1.8-4 | 2020-07-15 |
| later | 1.1.0.1 | 2020-07-15 |
| viridisLite | 0.3.0 | 2020-06-15 |
| SingleCellExperiment | 1.12.0 | 2020-10-28 |
| tibble | 3.1.1 | 2021-04-19 |
| pheatmap | 1.0.12 | 2020-07-16 |
| stats | 4.0.1 | 2020-06-07 |
| beeswarm | 0.2.3 | 2020-07-15 |
| IRanges | 2.24.1 | 2020-12-13 |
| fastICA | 1.2-2 | 2020-07-15 |
| bluster | 1.0.0 | 2020-10-28 |
| statmod | 1.4.35 | 2020-10-20 |
| ellipsis | 0.3.1 | 2020-07-15 |
| BiocStyle | 2.18.1 | 2020-11-25 |

---

**System Information**

|  | systemInfo |
| --- | --- |
| version | R version 4.0.1 (2020-06-06) |
| platform | x86\_64-apple-darwin17.0 (64-bit) |
| locale | en\_CA.UTF-8 |
| OS | macOS 10.16 |
| UI | X11 |

**Scikick Configuration**

```
cat scikick.yml
```

```
### Scikick Project Workflow Configuration File

# Directory where Scikick will store all standard notebook outputs
reportdir: report

# --- Content below here is best modified by using the Scikick CLI ---

# Notebook Execution Configuration (format summarized below)
# analysis:
#  first_notebook.Rmd:
#  second_notebook.Rmd: 
#  - first_notebook.Rmd     # must execute before second_notebook.Rmd
#  - functions.R            # file is used by second_notebook.Rmd
#
# Each analysis item is executed to generate md and html files, E.g.:
# 1. <reportdir>/out_md/first_notebook.md
# 2. <reportdir>/out_html/first_notebook.html
analysis: !!omap
- index.Rmd:
- notebooks/nestorowa/import.Rmd:
- notebooks/nestorowa/quality_control.Rmd:
  - notebooks/nestorowa/import.Rmd
- notebooks/nestorowa/normalization.Rmd:
  - notebooks/nestorowa/quality_control.Rmd
- notebooks/nestorowa/further_exploration.Rmd:
  - notebooks/nestorowa/normalization.Rmd
- notebooks/grun/import.Rmd:
- notebooks/grun/quality_control.Rmd:
  - notebooks/grun/import.Rmd
- notebooks/grun/normalization.Rmd:
  - notebooks/grun/quality_control.Rmd
- notebooks/grun/further_exploration.Rmd:
  - notebooks/grun/normalization.Rmd
- notebooks/paul/import.Rmd:
- notebooks/paul/quality_control.Rmd:
  - notebooks/paul/import.Rmd
- notebooks/paul/normalization.Rmd:
  - notebooks/paul/quality_control.Rmd
- notebooks/paul/further_exploration.Rmd:
  - notebooks/paul/normalization.Rmd
- notebooks/merged/merge.Rmd:
  - notebooks/grun/quality_control.Rmd
  - notebooks/paul/quality_control.Rmd
  - notebooks/nestorowa/normalization.Rmd
- notebooks/merged/combined_analysis.Rmd:
  - notebooks/merged/merge.Rmd
version_info:
  snakemake: 6.0.2
  ruamel.yaml: 0.16.12
  scikick: 0.2.1
# Optional site theme customization
output:
  BiocStyle::html_document:
    code_folding: hide
    theme: readable
    toc_float: true
    toc: true
    number_sections: false
    toc_depth: 5
    self_contained: true
```

---

**Functions**


  
  


Next (Project Map)


skmap


cluster\_/

/


cluster\_notebooks/nestorowa/

notebooks/nestorowa/


cluster\_notebooks/grun/

notebooks/grun/


cluster\_notebooks/paul/

notebooks/paul/


cluster\_notebooks/merged/

notebooks/merged/


notebooks/grun/quality\_control.Rmd


Quality Control


notebooks/merged/merge.Rmd


Merge


notebooks/grun/quality\_control.Rmd->notebooks/merged/merge.Rmd


notebooks/grun/normalization.Rmd


Normalization


notebooks/grun/quality\_control.Rmd->notebooks/grun/normalization.Rmd


notebooks/merged/combined\_analysis.Rmd


Combined Analysis


notebooks/merged/merge.Rmd->notebooks/merged/combined\_analysis.Rmd


notebooks/paul/quality\_control.Rmd


Quality Control


notebooks/paul/quality\_control.Rmd->notebooks/merged/merge.Rmd


notebooks/paul/normalization.Rmd


Normalization


notebooks/paul/quality\_control.Rmd->notebooks/paul/normalization.Rmd


notebooks/nestorowa/normalization.Rmd


Normalization


notebooks/nestorowa/normalization.Rmd->notebooks/merged/merge.Rmd


notebooks/nestorowa/further\_exploration.Rmd


Further Exploration


notebooks/nestorowa/normalization.Rmd->notebooks/nestorowa/further\_exploration.Rmd


index.Rmd


Index


notebooks/nestorowa/import.Rmd


Import


notebooks/nestorowa/quality\_control.Rmd


Quality Control


notebooks/nestorowa/import.Rmd->notebooks/nestorowa/quality\_control.Rmd


notebooks/nestorowa/quality\_control.Rmd->notebooks/nestorowa/normalization.Rmd


notebooks/grun/import.Rmd


Import


notebooks/grun/import.Rmd->notebooks/grun/quality\_control.Rmd


notebooks/grun/further\_exploration.Rmd


Further Exploration


notebooks/grun/normalization.Rmd->notebooks/grun/further\_exploration.Rmd


notebooks/paul/import.Rmd


Import


notebooks/paul/import.Rmd->notebooks/paul/quality\_control.Rmd


notebooks/paul/further\_exploration.Rmd


Further Exploration


notebooks/paul/normalization.Rmd->notebooks/paul/further\_exploration.Rmd


---
