# Supplementary material for: Scikick: A sidekick for workflow clarity and reproducibility during extensive data analysis
Source: PLoS One. 2023 Jul 27;18(7):e0289171. doi: 10.1371/journal.pone.0289171 (PMC10374128; doi:10.1371/journal.pone.0289171)
Supplement: Attachment — Submitted filename: response_to_the_reviewers_comments.docx [file pone.0289171.s002.docx]

***Reviewer #1:***

**Comment:** *Regarding the paper, I have struggled a little bit with figure 2. I was able to fully understand it after browsing the example report online and navigating through the different sections, so my suggestion is try improving the figure 2 legend so that readers can understand it easier. For instance, you could add a files tree of the project and briefly explain them.*

**Response:** We have taken the reviewers suggestion to better highlight the merits of Scikick being displayed in figure 2 by including the file tree and further describing the figure (L133-L139).

**Comment:** *I'd suggest authors adding a new section (and maybe a figure) to explain the basic commands of the "sk" tool (init, add, status and run) as it is important for readers to see how the tool works and how dependencies are defined.*

**Response:** In our initial submission, we have included detailed information regarding the execution in our supplemental information in an effort to reduce too much technicality in the manuscript. Based on the reviewer’s feedback, we have included a new section as an overview of the command-line interface (L94-L103), yet direct to the documentation for longer form tutorials and examples.

**Comment:** *Have you considered including tools or some mechanisms to obtain user-friendly readable diffs between notebook files? By default, "git diff" commands on Python notebooks are not very useful.*

**Response:** Thank you for the suggestion. While it is an interesting idea, we think it is beyond the scope of Scikick. There are some dedicated tools to specifically address this challenging issue (*e.g.,* https://nbdime.readthedocs.io/en/latest/), and we feel these specialty tools and others like it will work in tandem with Scikick.

**Comment:** *Regarding figure 3, what does mean "verified archive"? I think that this figure can be also improved. For me, the flow would go from local development to the GitLab server and from that point the execution of the "continuous analysis" triggered. With the current image, one can think that the continuous analysis is performed in the same computer as the workflow is being developed.*

**Response:** We have replaced “verified archive” in Figure 3 with “GitLab Repository” in order to avoid the possible misunderstanding pointed out by the reviewer. We also have clarified what we mean by “verified” throughout the revised manuscript (L160-L161; L188-L190).

***Reviewer #2:***

**Comment:** *Figure 1 seems a bit superfluous to me. It attempts to provide a high-level justification for the tool, but in my opinion, it doesn't add anything to the paper beyond what is already in the text.*

**Response:** We agree with the reviewer that it is somewhat repetitive to what is described in the main text. However, we believe that an overview may serve a similar role to graphical abstracts for those who are trying to rapidly process information, as a visual aid.

**Comment:** *The paper focuses mostly on justifying the need for the tool. But it provides little insight on *how* the tool works. For example, how does it collect the metadata and integrate everything? How does it integrate with snakemake? Etc. I don't think it's necessary to provide a lot more detail. But a little more would be helpful.*

**Response:** Much of this information was included as supplemental information (found in the documentation “core design”) or was custom code that would be trivial to explain (e.g. “sys.time” for logging runtime). We used the article to highlight the need for tools like Scikick and the utility they add (*i.e.*, the why rather than the how). We have made additions to the manuscript where appropriate to better allude to this content and to the underlying architecture of the software (L91; L109; L110; L112; L133-L136; L165-L166).

**Comment:** *The paper says, "Additionally, maintaining a simple project structure suitable for version control, further eases the adoption of Continuous Analysis on version control platforms (e.g., GitHub, GitLab, BitBucket, etc.)." I'm not sure if I understand exactly what this is saying. How does it support using version control?*

**Response:** This sentence combined too many concepts and has now been expanded for clarity (L165-L167).

**Comment:** *I'm probably missing it, but I don't see any explicit instructions about how to install the software. That's a pretty standard thing to see on documentation sites.*

**Response:** The installation instructions should have been found on the homepage (https://petronislab.camh.ca/pub/scikick). We have changed this link from “Start using Scikick” to “Installation and Usage” to avoid confusion and have added a “Quick-Start” installation to the GitHub homepage to further improve accessibility.
